# miR824/AGAMOUS-LIKE16 Module Integrates Recurring Environmental Heat Stress Changes to Fine-Tune Poststress Development

**DOI:** 10.3389/fpls.2019.01454

**Published:** 2019-11-25

**Authors:** Henrik Mihály Szaker, Éva Darkó, Anna Medzihradszky, Tibor Janda, Hsiang-chin Liu, Yee-yung Charng, Tibor Csorba

**Affiliations:** ^1^Agricultural Biotechnology Institute, NARIC, Godollo, Hungary; ^2^Faculty of Natural Sciences, Eötvös Lóránd University, Budapest, Hungary; ^3^Agricultural Institute, Centre for Agricultural Research, Hungarian Academy of Sciences, Martonvásár, Hungary; ^4^Agricultural Biotechnology Research Center, Academia Sinica, Taipei, Taiwan

**Keywords:** AGAMOUS-LIKE16, miR824, heat stress, posttranscriptional memory factor, FLOWERING LOCUS T

## Abstract

Plant development is continually fine-tuned based on environmental factors. How environmental perturbations are integrated into the developmental programs and how poststress adaptation is regulated remains an important topic to dissect. Vegetative to reproductive phase change is a very important developmental transition that is complexly regulated based on endogenous and exogenous cues. Proper timing of flowering is vital for reproductive success. It has been shown previously that AGAMOUS LIKE 16 (AGL16), a MADS-box transcription factor negatively regulates flowering time transition through FLOWERING LOCUS T (FT), a central downstream floral integrator. AGL16 itself is negatively regulated by the microRNA miR824. Here we present a comprehensive molecular analysis of miR824/AGL16 module changes in response to mild and recurring heat stress. We show that miR824 accumulates gradually in response to heat due to the combination of transient transcriptional induction and posttranscriptional stability. miR824 induction requires heat shock *cis*-elements and activity of the HSFA1 family and HSFA2 transcription factors. Parallel to miR824 induction, its target AGL16 is decreased, implying direct causality. AGL16 posttranscriptional repression during heat stress, however, is more complex, comprising of a miRNA-independent, and a miR824-dependent pathway. We also show that *AGL16* expression is leaf vein-specific and overlaps with miR824 (and FT) expression. *AGL16* downregulation in response to heat leads to a mild derepression of *FT*. Finally, we present evidence showing that heat stress regulation of miR824/AGL16 is conserved within *Brassicaceae*. In conclusion, due to the enhanced post-transcriptional stability of miR824, stable repression of AGL16 is achieved following heat stress. This may serve to fine-tune FT levels and alter flowering time transition. Stress-induced miR824, therefore, can act as a “posttranscriptional memory factor” to extend the acute impact of environmental fluctuations in the poststress period.

## Introduction

In nature, plants adapt to the diurnally and seasonally fluctuating environment for successful growth and reproduction. Heat stress (HS) is one of the most important abiotic stresses. Plant HS response (HSR) is triggered by a number of temperature sensing pathways (Mittler et al., 2012; Yeh et al., 2012). One important task of the HSR signal transduction pathways is the activation of HS transcription factors (HSFs) (Mittler et al., 2012; Scharf et al., 2012; Yeh et al., 2012). Multiple HSFs are involved in basal HSR, HSFA1 family transcription factors (in *Arabidopsis* HSFA1a, HSFA1b, HSFA1d, and HSFA1e) being its master regulators (Liu et al., 2011; Yoshida et al., 2011). HSFA1 paralogs, among others, turn on the transcription of HSFA2 (Charng et al., 2007; Nishizawa-Yokoi et al., 2011; Liu and Charng, 2012; Liu and Charng, 2013). HSFA1s, together with HSFA2 induce the expression of various types of heat shock proteins (HSPs) and nonchaperone proteins (Scharf et al., 2012). When plants encounter HS for the first time, they become acclimated (primed). Under natural conditions, acclimation occurs gradually during the day and repeatedly at the beginning of the hot season. This so-called acquired thermotolerance allows plants to survive upcoming stronger, even lethal stresses (Mittler et al., 2012; Lamke and Baurle, 2017; Liu et al., 2018). Active maintenance of acquired thermotolerance for several days after the stress is the HS memory. HSFA2 is the central component of HS memory (Charng et al., 2007; Lamke et al., 2016; Liu et al., 2018). HS memory also requires the chromatin remodeling factor FORGETTER1 (Brzezinka et al., 2016), the chromatin-associated protein BRUSHY1/TONSOKU/MGOUN3 (Brzezinka et al., 2018), the HSP HEAT-STRESS-ASSOCIATED32 (HSA32)(Charng et al., 2006; Wu et al., 2013), a peptidyl cis/trans isomerase ROTAMASE FKBP1(Meiri and Breiman, 2009) and HSFA1s or HSFA1-related factors (Liu et al., 2018). While the different forms of HSR have been intensively studied, how plants integrate sporadic or repeated stress signals and alter their development following stress is much less known.

MicroRNAs (miRNAs) are an important class of small RNAs, the central players of RNA silencing (Axtell, 2013; Rogers and Chen, 2013; Borges and Martienssen, 2015). miRNAs are encoded by distinct genes, transcribed by RNA polymerase II. miRNA transcripts may contain introns, therefore they undergo splicing. Subsequent to the splicing, the fold-back structures of miRNA precursors (pri-miRNAs) are maturated by DICER-LIKE proteins in two steps to give rise to the pre-miRNA and the mature miRNA duplex. The mature miRNAs are loaded into ARGONAUTE proteins, the effector of silencing, to form RNA-induced silencing complex (RISC). In plants, RISC cleaves destabilizes or represses translation of its target messenger RNAs (mRNAs) guided by the nucleotide sequence of the loaded miRNA (Chen, 2004; Brodersen et al., 2008; Rogers and Chen, 2013; Borges and Martienssen, 2015). miRNAs regulate developmental and metabolic processes like cell differentiation, organ development, senescence, hormonal biosynthesis, nutrient uptake, and allocation (Schommer et al., 2008; Rubio-Somoza and Weigel, 2011; Matthewman et al., 2012; Luo et al., 2013; Li and Zhang, 2016). miRNAs are also involved in responses to environmental changes (Sunkar et al., 2012; Guan et al., 2013; Cui et al., 2014; Kruszka et al., 2014; Kumar, 2014; Zhang, 2015). Several miRNAs were shown to be heat-responsive in numerous species including *Arabidopsis thaliana*, *Brassica rapa*, *Populous euphratica*, *Triticum aestivum*, *Oryza sativa*, and others (Xin et al., 2011; Chen et al., 2012; Yu et al., 2012; Barciszewska-Pacak et al., 2015; Kumar et al., 2015; Mangrauthia et al., 2017; Gyula et al., 2018). In a few cases, the activity of stress-regulated miRNAs was studied in details and the precise molecular function unraveled (Guan et al., 2013; Cui et al., 2014; Stief et al., 2014; Ma et al., 2015; He et al., 2018).

MADS-box containing proteins are a large class of eukaryotic transcriptional factors involved in diverse pathways like development and environmental interactions (Yanofsky et al., 1990; Messenguy and Dubois, 2003; Smaczniak et al., 2012). In plants, MADS-box proteins play central and conserved roles in cell differentiation of the embryo, the gametophyte and the vegetative tissue development, the transition to flowering, the flower organogenesis, and fruit ripening (Yanofsky et al., 1990; Ferrandiz et al., 2000a; Ferrandiz et al., 2000b; Liljegren et al., 2000; Pelaz et al., 2000; Nesi et al., 2002; Michaels et al., 2003; De Bodt et al., 2005; Tao et al., 2012; Csorba et al., 2014; Fernandez et al., 2014; Whittaker and Dean, 2017). Several MADS-box proteins were shown to be stress-regulated (Lozano et al., 1998; Arora et al., 2007; Tardif et al., 2007; Saha et al., 2015; Chen et al., 2016). AGAMOUS-LIKE16 (AGL16) is a MIKC^C^-type MADS-box protein-coding gene, a member of the AGL17 clade (Smaczniak et al., 2012). The genes of the AGL17 clade are primarily expressed in roots suggesting root-related functions (Alvarez-Buylla et al., 2000; Burgeff et al., 2002; Gan et al., 2005). AGL16 is expressed more ubiquitously: besides root, it was found also in the stem and rosette leaves, inflorescence, and young siliques (Alvarez-Buylla et al., 2000). A miRNA, namely the miR824, negatively regulates *A. thaliana* AGL16 (Kutter et al., 2007; Hu et al., 2014). Two functions of AGL16 have been described in detail so far. Mutation of AGL16 or overexpression of miR824 decreased the number of higher-order stomata complexes, while the expression of miR824-resistant AGL16 in transgenic plants increased the incidence of higher-order stomata complexes (Kutter et al., 2007). The miR824/AGL16 module was also shown to modulate flowering time in *A. thaliana* under long-day conditions (Hu et al., 2014; McClung et al., 2016). The AGL16 protein interacts directly with the SHORT VEGETATIVE PHASE protein and indirectly with FLOWERING LOCUS C (FLC). FLC is a central regulator of flowering transition (Lee and Amasino, 1995; McClung et al., 2016; Whittaker and Dean, 2017). The FRIGIDA (FRI) complex drives high expression of FLC (Geraldo et al., 2009; Choi et al., 2011). The negative regulatory effect of AGL16 on the flowering time is fully dependent on the repression of FLOWERING LOCUS T (FT) (Hu et al., 2014; Romera-Branchat et al., 2014). *rsa*-miR824, the *Raphanus sativus* homolog of miR824, was also linked to flowering regulation (Nie et al., 2015) suggesting functional conservation of the module. The presence of AGL16-like proteins in all the investigated plant species so far, suggests that they play important roles in angiosperm development and evolution (Becker and Theissen, 2003; Gramzow and Theissen, 2015).

In this work, we gather evidence suggesting that miR824 may act as an integrator of repeated HS signals to modulate AGL16 levels. As the impact of miR824-dependent AGL16 downregulation is primarily manifested poststress, miR824 may act as a posttranscriptional stress memory factor to alter development through fine-tuning FT pathway in response to environmental changes. We also show that heat-mediated regulation of the miR824/AGL16 module is conserved in multiple members of *Brassicaceae*.

## Materials and Methods

### Plant Material and Growth Conditions

*Arabidopsis* seeds were bleach-sterilized, stratified for 2 days in dark then plated on Murashige and Skoog (Duchefa M0222, https://www.duchefa-biochemie.com) medium agar plates (0.5 x Murashige and Skoog salts, 1% agar, pH 5.7). Plants were routinely grown in a Sanyo MLR-350 growth cabinet under cool white light at 21°C long day condition (16 h light/8 h dark photoperiod).

### Heat Stress Treatments

ACC: Gradient acclimation was done in the presence of light in a water bath in the course of 4 h: the temperature was rose starting at Zeitgeber Time ZT4 and reached 37°C at ZT7; plants were kept on 37°C for 1 h from ZT7 to ZT8. Plants were cooled back to 21°C following each treatment. Single ACC treatment was done on day 6, in case of two acclimations (ACCx2) on days 5 and 6, in case of three acclimations (ACCx3) on days 4, 5, and 6 postgermination. Samples were taken on day 6 immediately after treatment for ACC samples, or the next day at ZT8 for REC samples.

HS: for direct HS naïve 7 day old seedlings grown on agar plates were exposed to 45°C in a water bath in the presence of light for 30 min and samples collected immediately at midday (ZT8). ACCx3 + HS: seedlings were first acclimated as described for ACCx3 then exposed to HS. 37°Cx3: seedlings were exposed to direct 37°C in a water bath in the presence of light for 1 h at ZT7 to ZT8, then cooled back to 21°C. Treatments were done on days 4, 5, and 6 postgermination. Samples were taken at ZT8.

For FT measurements seedlings were heat-treated at 37°C for 1 h each (from ZT7 to ZT8) at days 7, 8, and 9. Samples were taken at the end of the light period (ZT16) on day 10 (Hu et al., 2014) to allow recovery of *AGL16* in Col-*FRI;∆824* plants.

### Accession Numbers of Genes Used in the Study

miR824 (At4g24415), miR398a (At2g03445), U6 (At3g14735), AGL16 (At3g57230), ACT2 (At3g18780), PP2a (At1g69960), HSFA1a (At4g17750), HSFA1b (At5g16820), HSFA1d (At1g32330), HSFA1e (At3g02990), HSFA2 (At2g26150), HSFA3 (At5g03720), HSFA6a (At5g43840), HSFA6b (At3g22830), HSFA7a (At3g51910), HSFA7b (At3g63350), FRIGIDA (At4g00650), FLC (At5g10140), Bna-miR824 (LOC106440800), BnaAGL16 (LOC106357131), BnaPP2A5 (LOC106382560), XRN4 (At1g54490), SKI2 (At3g46960), CSD1 (At1g08830), PP2AA3 (At1g13320), RD29A (At5g52310), UBC22 (At5g05080).

### Mutant and Transgenic Lines Used in the Study

*agl16-1* (SALK_104701) (Kutter et al., 2007), ∆*824* (SALK_138988), *MIM824* [line 12 from (Hu et al., 2014)], *aTK*, *bTK*, *dTK*, *eTK*, and *QK* (Liu et al., 2011), *hsfa2* (SALK_008978) (Charng et al., 2007), *hsfa3* (SALK_011107) (Schramm et al., 2008), *hsfa6a* (SALK_089880) (Hwang et al., 2014), *hsfa6b* (GK_513_A02) (Huang et al., 2016), *hsfa7a* (WiscDsLox318F08), *hsfa7b* (SALK_152004) (Charng et al., 2007), *xrn4-6* (SALK_014209) (Gy et al., 2007), *ski2-2* (SALK_129982) (Branscheid et al., 2015), *flc-2* (Michaels and Amasino, 1999).

### Genotyping

Genomic DNA was extracted with extraction buffer [100 mM glycine, 10 mM ethylenediaminetetraacetic acid (EDTA), 100 mM NaCl, 2% sodium dodecyl sulfate (SDS)] at room temperature, purified with phenol:chloroform: isoamyl alcohol (25:24:1) pH 8.0, precipitated in ethanol and resuspended in sterile water. Genotyping PCR was done using DNA Taq polymerase (NEB, M0273S) based on manufacturer instructions. For primer sequences see **Primer Table**.

### Transgene Constructs

For miR824-promoter GUS transgenic lines, the 2,954 bp fragment comprising the 2,852 bp sequences upstream of the transcription start site (TSS, +1) and 102 bp segment downstream of TSS was amplified in a PCR reaction (Phiuson, Thermo Scientific) cloned into the pGEM-T-easy vector (Promega). For mutant promoter generation PCR mutagenesis was done using mutagen primers (for sequences see primer Table) (wt HSE1: gTTCtaGAAc, mutant HSE1: gTCtaGAc, wt HSE-like 2: cTTCaaaGAAt, mutant HSE-like 2: cTTaaaAAt, wt HSE-like 3: aTTCaaGGAg, mutant HSE-like 3: aTTaaGAg), and then fused with the GUS reporter gene in pCAMBIA1301 at EcoRI and NcoI sites following elimination of 35S promoter region. After sequencing the inserted fragments to confirm the absence of mutations caused by PCR and the presence of site-directed mutations introduced in the mutant HSE promoters, independent transgenic lines were generated in Col-0 *via* floral dipping using C58C1 *Agrobacterium* strain (Clough and Bent, 1998). Plants were selected on hygromycin and confirmed as positives in Northern blot and GUS activity assays. The first rosette leaf of T1 seedlings or mature leaves, stems, and inflorescence of T1 plants was used for Northern blotting, qRT-PCR or GUS staining assays. Northern blotting, qRT-PCR assays, and GUS staining were done in at least three biological replicates if not stated otherwise.

For *pAGL16::GUS* reporter construct promoter sequence of AGL16 gene comprising 930 bp upstream of the TSS and 1,892 bp downstream of TSS (comprising of the 5′ untranslated region, first exon, first intron, and second exon, see also **Figure S4A**) was PCR amplified (Phiuson, Thermo Scientific) and cloned in-frame with GUS ORF into pCAMBIA1301-EcoRI/NcoI (EcoRI-35S promoter–NcoI fragment was previously eliminated from the vector). Cloning was done using the Gibson Assembly method (https://sgidna.com). After sequencing the inserted fragments to confirm the absence of mutations caused by PCR, independent transgenic lines were generated in Col-0 *via* floral dipping using C58C1 agrobacterium strain.

For primers please see **Supplementary Materials**.

### Generation of QK;*phsfa1a*::*HSFA1a-3xHA* Plants

To generate the transgenic line expressing a C-terminally 3xHA-tagged HSFA1a in *QK* background, the genomic DNA of *Arabidopsis* (Col-0) *HSFA1a* comprising the 754 bp sequences upstream of TSS and the full CDS was amplified in a PCR reaction, then fused to a 3xHA coding sequence and the NOS terminator in a binary vector. The construct was then transferred into the *Agrobacterium* GV3101 strain and transformed into the *QK* mutant as previously described (Liu and Charng, 2013).

### GUS Staining

Plant material was incubated for 30 min in 90% (v/v) acetone on ice, rinsed with 50 mM sodium phosphate buffer, pH 7.0, and incubated overnight at 37°C in staining solution (0.5 mg/ml X-Gluc [5-bromo-4- chloro-3-indolyl-b-D-glucuronide], 50 mM sodium phosphate buffer, pH 7.0, 0.5 mM potassium ferrocyanide, 0.5 mM potassium ferricyanide, and 0.1% [v/v] Triton X-100). After staining, samples were washed with 50 mM sodium phosphate buffer, pH 7.0, and cleared in 70% (v/v) ethanol. The GUS histochemical staining was visualized under a light stereomicroscope (Leica MZ10 F). For each transgenic construct multiple independent lines were assayed (see figure legends).

### RNA Extraction and Northern Blotting

Total RNA was extracted from approximately 30 mg seedlings. The homogenized plant materials were resuspended in 600 µl of extraction buffer (0.1 M glycine-NaOH, pH 9.0, 100 mM NaCl, 10 mM EDTA, 2% SDS) and mixed with an equal volume of phenol pH 4,3. The aqueous phase was treated with equal volumes of phenol-chloroform and chloroform, precipitated with ethanol and resuspended in sterile water. RNA gel blot analysis of higher molecular weight RNAs was performed as described previously (Silhavy et al., 2002).

RNA gel blot analysis of 21–24 nt RNAs was performed as follows. Approximately 5 µg of total RNA was separated by 15% polyacrylamide gel electrophoresis (PAGE) with 8.6 M urea and 1x Tris–borate–EDTA. RNA was electroblotted onto Hybond-NX membranes and fixed by chemical crosslinking at 60°C for 1 h (Damm et al., 2015). Small RNA Northern blot hybridization and analysis were performed using complementary DNA oligo for miR824 and miR824-*3p* or locked nucleic acid oligonucleotides for miR159 and miR398a (Exiqon, http://www.exiqon.com).siRNA Northern blotting assays were done in at least three biological replicates if not otherwise stated.

### qRT-PCR

For qRT-PCR assays, 5 µg total RNA was DNase treated according to manufacturer's instructions (Ambion AM2222, www.thermofisher.com), precipitated in ethanol, resuspended in sterile water. One microgram of DNase-treated total RNA and random primer was used for the first-strand complementary DNA reaction according to the manufacturer's instructions (NEB, E6300S, www.neb.com). qPCRs were done using qPCR Master Mix (NEB, M3003S, www.neb.com) according to the manufacturer's instructions. qPCR reactions were run in a Light Cycler 96 (Roche) Real-Time PCR machine. Samples were collected at Zeitgeber Time 8 (ZT8). At least three biological samples were assessed in each experiment and standard error bars shown. P values were calculated using unpaired two-tailed Student t-test to assess the significance of differences. For primers please see **Supplementary Materials**.

FT mRNA measurements: to avoid derepression of FT by high ambient temperatures, instead of ACCx3 we heat-treated plants by exposing them directly to 37°C for 1 h and cooling back immediately to 21°C. Treatment was applied repeatedly for 3 days in a row (37°C x 3). This dose of moderate HS does not affect the overall growth and survival of the seedlings (Stief et al., 2014). For AGL16 change detection, we collected samples at midday (ZT8), while as *FT* peaks at the end of the light period we collected samples at the end of the light period (ZT16).

### Western Blotting

Seedlings were homogenized in extraction buffer (150 mM Tris–HCl, pH 7.5, 6 M urea, 2% SDS, and 5% µ-mercaptoethanol). Samples were boiled, and cell debris was removed by centrifugation at 18,000×*g* at 4°C for 10 min. The supernatants were resolved on 12% SDS polyacrylamide gel electrophoresis, transferred to Hybond PVDF membranes (GE Healthcare) and subjected to Western blot analysis. For detection 3xHA-tagged HsfA1a, horseradish peroxidase conjugated antibody (Roche, 3F10) was used. The proteins were visualized by chemiluminescence (ECL kit; GE Healthcare) according to the manufacturer's instructions.

### Chromatin Immunoprecipitation

ChIP assays were performed using pooled 10-day-old heat-treated seedlings. ChIP experiments were done as described (Angel et al., 2011), using the anti-HA Affinity Matrix (Roche, 11815016001). The ChIP data were quantified by qPCR. ACTIN2 was used as an internal reference gene. For primers please see **Supplementary Materials**.

### Physiological Measurements

#### Measurements of Gas Exchange Analyses Under Different Temperature Conditions

The gas exchange analysis was performed on intact attached leaves of 21-day old plants using a Ciras 3 portable photosynthesis instrument equipped with a narrow (1.7 cm^2^) leaf cuvette (PP systems, Haverhill MA, United States). For ACCx3 pretreated plants treatments were done on days 18, 19, 20. The net assimilation rate (Pn), stomatal conductance (gs), and transpiration rate (E) were determined under two temperature conditions (22 and 37°C) and at steady state of photosynthesis using a CO_2_ level of 400 µl l^−1^ and light intensity of 700 µmol m^−2^ s^−1^.

#### Temperature-Dependent Chlorophyll a Fluorescence Measurements

The heat-induced changes of chlorophyll *a* fluorescence parameters were also detected on intact detached leaves by the use of a pulse amplitude modulated fluorometer (Imaging-PAM M series, Walz, Effeltrich, Germany) completed with a thermoregulatory instrument consisting of a water-cooled Peltier thermoelectric module, a thermocouple thermometer, and a control unit. The measurements were started at 21°C and after the photosynthesis was steady (15 min) under actinic light illumination at 100 µmol m^−2^ s^−1^ the temperature was increased from 21 to 55°C at a rate of 1°C min^−1^. During the measurements, 1.0 s saturated flashes (photosynthetic photon flux density = 3,000 µmol m^−2^ s^−1^) provided by an LED-Array Illumination Unit IMAG-MAX/L (λ = 450 nm) were applied at each degree Celsius. The effective quantum yield of PS (II) parameter was shown.

### *In situ* Hybridization

*In situ* hybridization was done as described before (Medzihradszky et al., 2014). For probe preparation, AGL16 or PIN1 complementary DNA was cloned into the pBSK+ vector. RNA probe was prepared using T7 *in vitro* transcription, based on manufacturer's instructions (Thermo). As AGL16 antisense had a high background and *agl16-1* negative control could not be used (since contains *AGL16-T-DNA* transcript) we used PIN1 antisense RNA as a negative control (**Figure 5B**). PIN1 mRNA is not expressed in leaf veins but is strongly expressed in shoot apical meristem.

### Large Datasets: RNA Transcriptome Analysis

Total RNA samples of 7 days old Col-0 and *agl16-1* seedlings have been prepared for Illumina sequencing (in four biological replicates each). Paired-end library preparation was done using TruSeq Stranded mRNA LT Sample Prep Kit according to TruSeq Stranded mRNA Sample Preparation Guide (Part # 15031047 Rev. E). Raw RNAseq data have been made available in the SRA repository (SRP151884). RNA reads were aligned to the *Arabidopsis* genome (TAIR10) (Lamesch et al., 2012) using hisat2-2.1.0 (Kim et al., 2015). Bedgraph files have been generated using SAMtools 1.8 (Li et al., 2009) and deepTools2 (Ramirez et al., 2016) and visualized by Integrated Genome Browser (v9.0.0) strand specifically (Freese et al., 2016).

## Results

**Figure 1:**
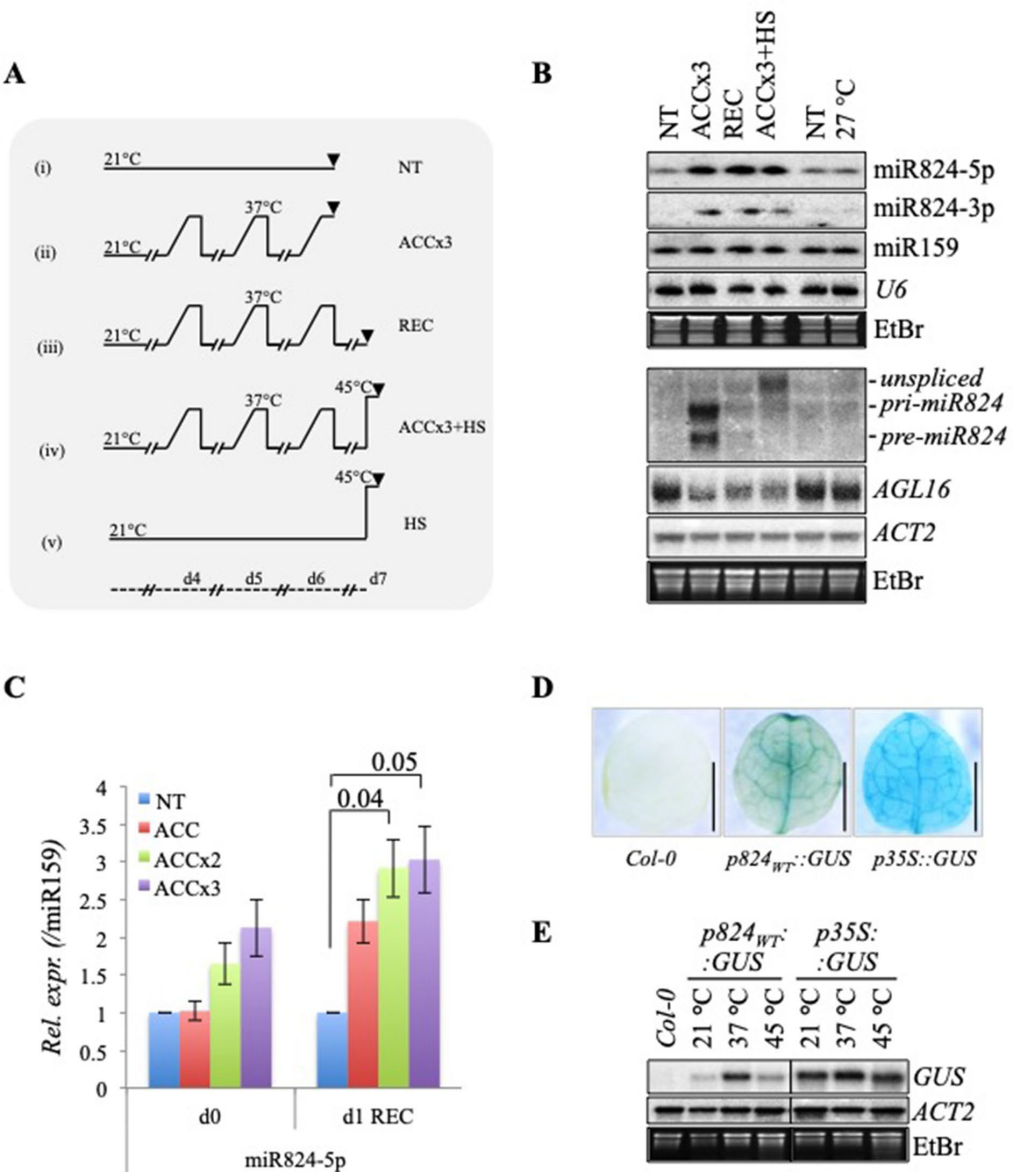
Recurring gradient acclimation causes the accumulation of miR824 and stable depletion of *AGL16* messenger RNA (mRNA). **(A)** Heat stress regimes used in the study. (i) nontreated, NT; (ii) acclimation repeated three times, ACCx3: heat gradient elevated in the course of 4 h; (iii) 1 day recovery following ACCx3 treatment, REC; (iv) heat stress following acclimation, ACCx3 + HS; (v) direct heat stress, HS; temperatures are shown on top of each regime, timeline is shown on bottom, samples were all collected at midday; **(B)** *AGL16*, miR824, and *miR824-precursor* changes in response to the treatments depicted in (A); miR824-5p and -3p accumulates following repeated gradient acclimation and remains high during a day recovery. miR824 precursor forms (*unspliced*, *pri-miR824*, and *pre-mir824*) are transiently induced by heat but not 27°C. High temperatures cause a persistent decrease in *AGL16*; treatments are shown on the top; **(C)** miR824 gradually accumulates in response to recurring heat: acclimation repeated once, twice or three times (ACC, ACCx2, or ACCx3); 1 day recovery, (+1d REC) (quantification of Northern blot data; bars represent standard errors based on three biological replicates; p values based on two-tailed Student's *t*-test, NT value was set to 1.); **(D)** GUS staining of the first rosette leaf of 2-week-old Col-0 control plant, and plants transformed with *p824_WT_::GUS* or *p35S::GUS* control reporter constructs. **(E)** miR824 promoter-driven GUS transcription is induced by heat treatment in *p824_WT_::GUS* transgenic plants; Col-0 and *p35S::GUS* plants were used as controls; *ACTIN2* (*ACT2*) and ethidium-bromide staining (EtBr) are shown as loading controls.

miRNAs are 10 times more stable than mRNAs on average, having a lifespan of several days (Csorba et al., 2010; Gantier et al., 2011; Sanei and Chen, 2015). We hypothesized that due to their high stability, the stress-responsive miRNAs might gradually accumulate in response to recurring stress cues and act as lasting memory factors to fine-tune their targets on longer terms after stress. To find miRNAs responsive to heat and potentially involved in HS adaptation, we treated plants using an HS program that mimics natural conditions (Mittler et al., 2012; Ling et al., 2018) (**Figure 1A**). Gradual treatments were applied by elevating temperature from 21 to 37°C in the course of four hours for three days in a row (acclimation treatment, ACCx3) (see also *Materials and Methods*). To understand how stable the changes are caused by ACCx3 treatment, we monitored the plants 1 day after acclimation (recovery sample, REC). We also subjected another batch of plants to HS 1 day after acclimation (ACCx3 + HS).

### Recurring Heat Stress Causes Accumulation of miR824 and Stable Decrease of *AGL16* mRNA Level

By applying heat acclimation repeatedly, among others, we have found miR824-5p and miRNA star strand miR824-3p to accumulate (**Figures 1B, C**). Stepwise accumulation of miR824-5p could be clearly observed when plants were exposed to an increasing number of ACC treatments (**Figures 1B, C**). miR824-5p and -3p were maintained at high levels after treatment (**Figures 1B, C**, recovery samples), (hereafter miR824-5p will be referred to as miR824).

To find out whether miR824 accumulation is due to transcriptional induction, we checked the level of its precursors. All spliced forms, including *pri-miR824* and *pre-miR824*, were found at elevated levels in the ACCx3 but dropped to the background the next day (**Figure 1B**, middle panel). The fast turnover of precursors is most likely due to the quick splicing and dicing processes. Higher levels of *unspliced-miR824* RNA were observed when plants were exposed to 45°C following acclimation (ACCx3 + HS, **Figure 1B**). This is likely caused by the stress-induced transcription coupled with inefficient splicing of nascent transcripts known to occur during HS (Ling et al., 2018). *miR824, -3p* or *precursors* did not accumulate in plants grown at elevated ambient temperatures (7 days at 27°C, **Figure 1B**). This finding was also confirmed by studying small RNA (sRNA) deep sequencing data published before (Gyula et al., 2018). miR824 transcriptional induction, therefore, is a *bona fide* stress response.

To confirm that miR824 is induced transcriptionally by heat, we created β-glucuronidase (GUS) reporter lines driven by the miR824 promoter (*p824* *_WT_* *::GUS*) (**Figures 1D, E** and **Figure S1**). As a control, we used *p35S::GUS* lines. The GUS activity was detected in the vasculature of leaves, stomata guard cells and trichomes as reported earlier (Hu et al., 2014). Besides these, GUS staining was observed in the actively dividing tissues including the root apical meristem, calyptra, veins and branching points, the shoot apical meristem, the flowering stem branching points, the tip of the siliques, and the veins of the floral organs (**Figure S1A**). We analyzed the HS-responsiveness of the miR824 promoter by monitoring GUS mRNA (**Figure 1E** and **Figure S1B**): *GUS* mRNA was strongly elevated at 37°C and slightly increased at 45°C (**Figure 1E**). GUS transcription driven from p35S promoter (*p35S::GUS*) was very mildly affected (**Figure 1E** and **Figure S1B**). These findings suggest that recurring, moderately high temperature (37°C) spells lead to the gradual accumulation of mature miR824 through the combination of repeated transient transcriptional activation and stability of the miRNA after stress. miR824, therefore, may integrate transient or sporadic HS signals that are reflected in its cumulated levels (**Figure 1C**).

**Figure 2:**
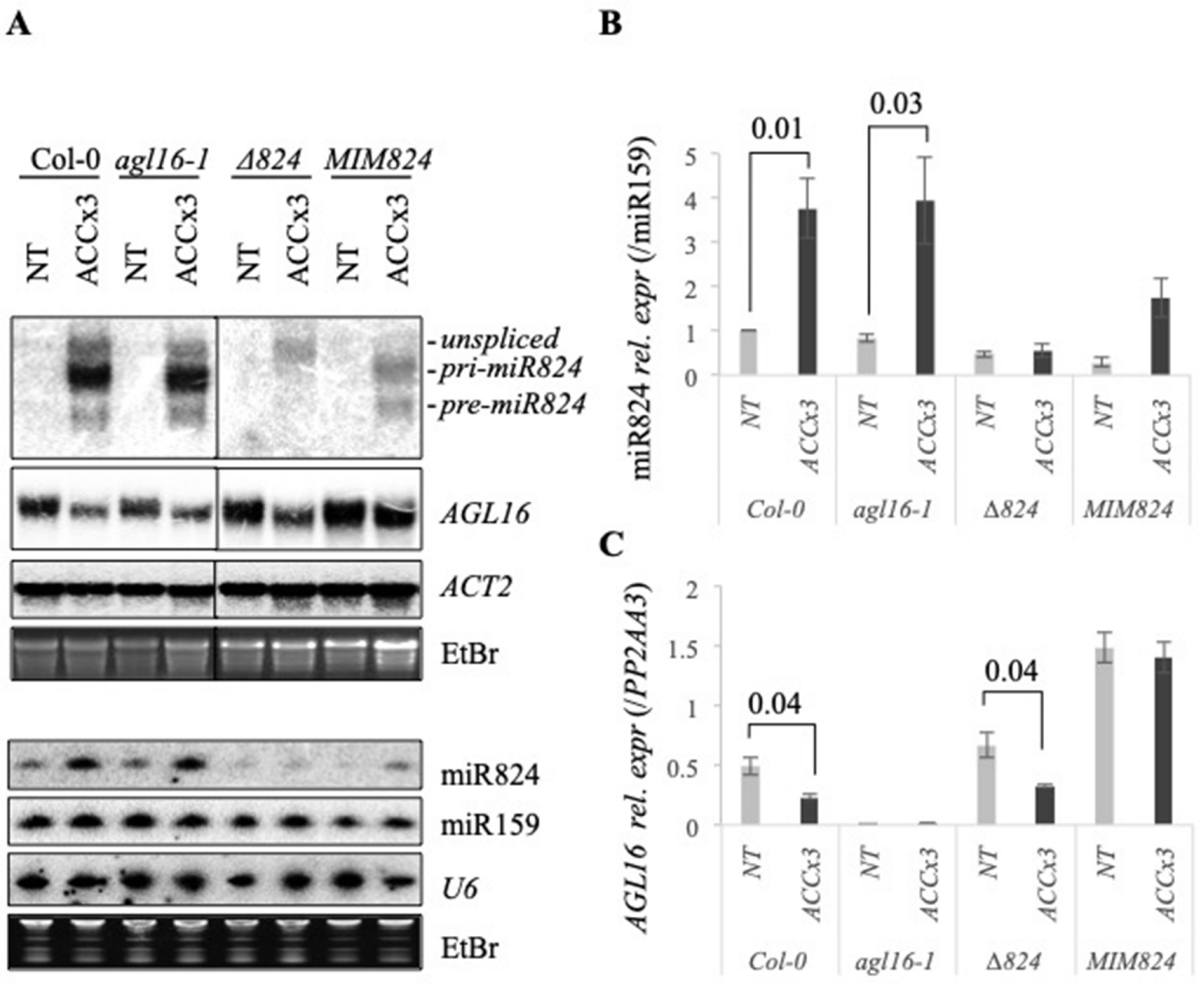
Heat-induced changes of miR824 precursors, miR824, and *AGL16* messenger RNA (mRNA) in the different miR824/AGL16 module mutants. **(A)** Northern blot assays showing *miR824-precursors*, miR824 and *AGL16* mRNA levels in Col-0 wild type, *agl16-1*, miR824-mutant (*∆824*), or miR824 mimicry (*MIM824*) plants; (nontreated, NT; acclimated three times, ACCx3); miR159, U6, *ACTIN2*, and ethidium-bromide (EtBr) staining are shown as loading controls. **(B)** Quantification of miR824 Northern blot signals in the different mutant backgrounds; bars represent standard errors based on three biological replicates; Col-0 NT value was set to 1. **(C)** Quantitative real-time PCR quantification of *AGL16* mRNA relative expression in the different mutants; bars represent standard errors based on three biological replicates; p values based on two-tailed Student's *t*-test.

Parallel to the miR824 accumulation, the miR824 target *AGL16* mRNA was stably depleted (**Figure 1B**). To understand how the heat-induced *AGL16* downregulation relates to the *AGL16* levels in the AGL16 mutant (*agl16-1*) or the miR824 mutant plants, we analyzed Col-0, *agl16-1* (SALK_104701), *∆824* (SALK_138988), and a previously characterized miR824-mimicry *MIM824* (Hu et al., 2014) lines during NT and ACC treatments (**Figure 2**). Downregulation of the *AGL16* mRNA under elevated temperatures was very efficient as it reached similar levels compared to the *agl16-1* mutant (**Figure 2A**). Heat-induced *AGL16* changes were confirmed by quantitative real-time PCR (qRT-PCR) as well (**Figure 2C**).

A clear signal was detected in the *agl16-1* plants by Northern blot (**Figure 2A**). The T-DNA insertion in *agl16-1* is within the last exon that may give rise to a truncated transcript. Besides qRT-PCR analysis (**Figure 2C**) we confirmed this by genotyping (**Figure S2A**) and RNAseq analysis (**Figure S2B**); (for the remnant signal of *AGL16*mRNA detected by Northern blot in *agl16-1* plants please see **Supplementary Information**). The *pri-miR824* induction and miR824 accumulation were not affected by the *agl16-1* mutation (**Figures 2A, B**).

We analyzed miR824 and *AGL16* mRNA levels in the miR824 mutants (*∆824* and *MIM824*) as well. In the *∆824* mutant the T-DNA insertion disrupts the MIR824 gene (located within the *pri-miR824* but not the *pre-miR824* transcript region). Induction of the miR824 transcription was detected during heat treatment (unspliced), but the *pri*- and *pre-miR824* maturation was largely impaired (**Figure 2A**). In spite of this, a residual amount of mature miR824 accumulates, and the level of *AGL16* mRNA is moderately increased in the *∆824* plants (nonsignificant vs. Col-0, **Figures 2A, C**). The residual amount of miR824 in the *∆824* plants, therefore, is enough to limit *AGL16* mRNA levels very efficiently. Heat-induced *AGL16* mRNA reduction was not altered in the *∆824* plants suggesting the involvement of a miR824-independent mechanism (**Figures 2A, C**).

In the *MIM824* line, a much stronger increase of *AGL16* mRNA could be observed (three-fold, **Figures 2A, C**) in agreement with earlier data (Hu et al., 2014). The MIR824 transcriptional induction and the mature miR824 accumulation were decreased in the *MIM824* plants. Although the mature miR824 accumulates (**Figures 2A, B**), its activity is efficiently neutralized by the presence of the target mimicry transcript RNA (Franco-Zorrilla et al., 2007; Hu et al., 2014) that leads to strong accumulation of AGL16. In *MIM824* the impact of heat on the expression of *AGL16* was limited suggesting a miR824-dependent and miR824-independent complex regulation (**Figure 2C**).

As *agl16-1* and *MIM824* plants were both shown to have a clear physiological phenotype (Kutter et al., 2007; Hu et al., 2014), heat stress induction of miR824 and downregulation of *AGL16* may play a role in HS adaptation.

### A Dual Mechanism for *AGL16* Downregulation During Heat Stress

As miR824 was proved to regulate *AGL16* directly and negatively (Kutter et al., 2007; Hu et al., 2014), and because the expression of miR824 and *AGL16* anticorrelated during HS a direct causality was suspected. Strikingly, we observed a decrease of *AGL16* expression during early HS, when the induction of miR824 was not yet detectable (direct 45°C for 30 min, **Figure S3A**). This suggests that the decrease of *AGL16* expression at high temperatures is miR824-independent. This is also corroborated by the *AGL16* dynamics in the miR824-mutants (*∆824* and *MIM824*) during HS (**Figure 2C**) and suggests a complex mechanism.

**Figure 3:**
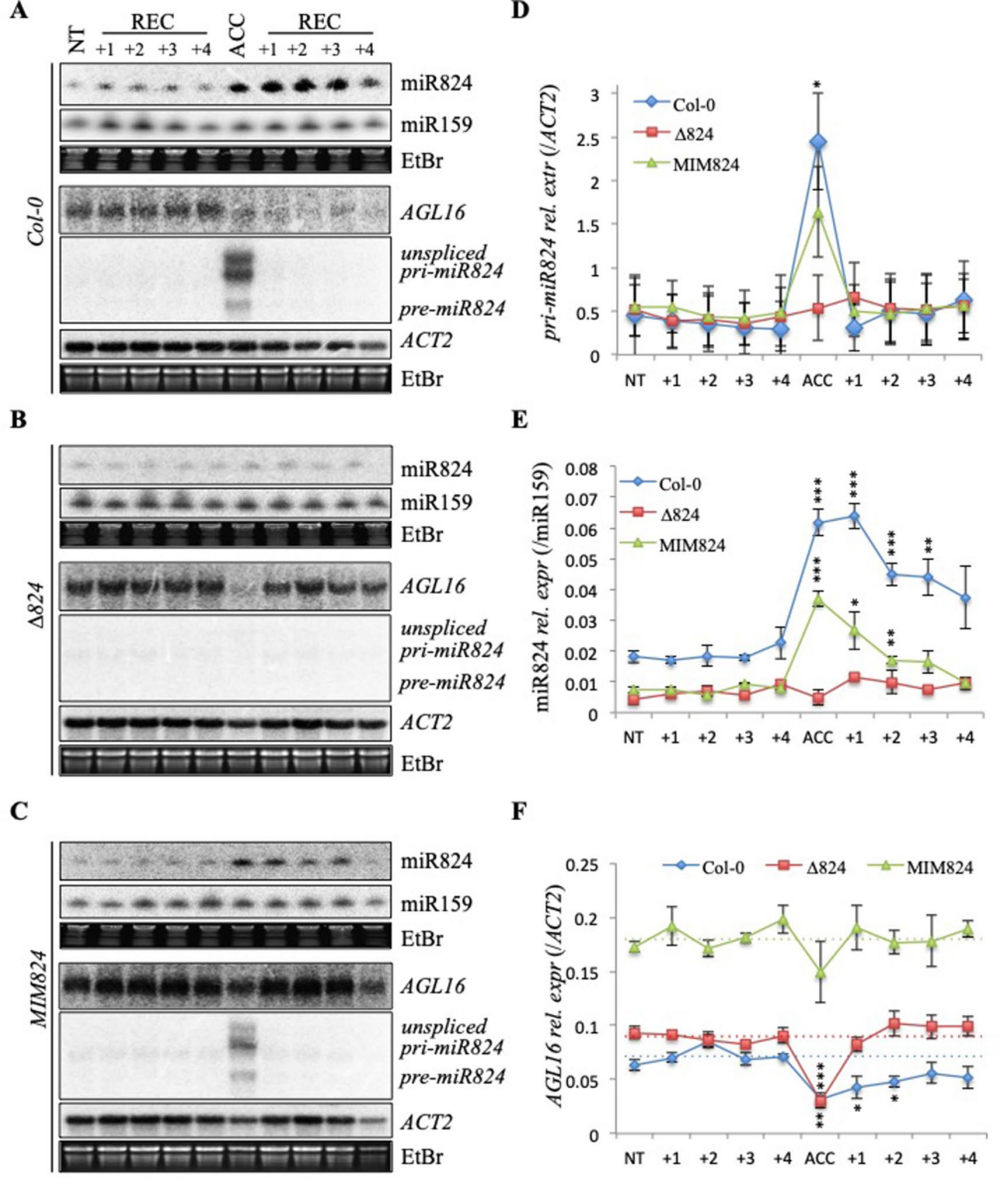
Dual depletion of *AGL16* messenger RNA (mRNA) during and following heat stress (HS). **(A**–**C)** Levels of *miR824-precursor*, mature miR824, and *AGL16* RNA changes during a time-course following a single treatment (ACC) or nontreated control (NT) (treatments are shown on the top: +1, +2, +3, +4 denote days passed after the treatment); **(A)** Col-0 plant samples, **(B)** miR824-mutant *∆824* plant samples and **(C)** miR824-mimicry *MIM824* plant samples; miR159, *ACTIN2*, and EtBr staining are shown as loading controls; **(D**–**F)** Quantification of at least three sets of Northern blot measurements: **(D)** *pri-miR824* data, **(E)** mature miR824 data, and **(F)** *AGL16* mRNA data; quantification data were first quantified to miR159 or *ACTIN2* controls than to nontreated controls; dotted lines represent basal level of *AGL16* in the wild-type and miR824-mutants; bars represent standard errors of three biological replicates; p values based on two-tailed Student's *t*-test (*p < 0.05, **p < 0.01, ***p < 0.001).

To separate the miR824-dependent and miR824-independent downregulation of *AGL16* during HS, we analyzed its mRNA levels in the miR824-defective plants. We compared *AGL16* downregulation and recovery during 4 days following a single ACC treatment in Col-0, *∆824*, and *MIM824* plants (**Figure 3**). A single ACC treatment was applied to achieve a transient induction, so we could monitor the half-life and the lasting activity of the mature miR824. The time scale was chosen because the sRNA-loaded RISCs were shown to be stable and active for several days (Csorba et al., 2010). The *pri-miR824* transcription and the miR824 accumulation were efficiently induced by the single ACC treatment in Col-0 plants, to a lower extent in *MIM824* plants but not in *∆824* mutant (**Figures 3A–E**). The *AGL16*mRNA levels immediately dropped in all genotypes suggesting a miR824-independent mechanism (**Figure 3F**). In the recovery period, the *AGL16* downregulation was maintained only in the Col-0 plants (**Figures 3A, F**); in the *∆824* mutant (where only a low level of miR824 is present) or *MIM824* plants (where miR824-RISC is inactive), the *AGL16* level was quickly restored by the next day (**Figures 3B, C, and F**). These findings show that the *AGL16* downregulation is caused by a miR824-dependent and a miR824-independent mechanism. On the other hand, these findings also suggest, that the miR824-dependent pathways' effect could be two-sided: it may contribute to the immediate downregulation of its target and can serve to keep it repressed following the acute stress period.

Next, we aimed to understand the miR824-independent decrease of *AGL16*. The mRNA abundance depends both on the rate of RNA transcription and RNA decay. To unpick these routes, we assayed *AGL16 unspliced* mRNA (*uAGL16*) levels as a proxy for transcriptional changes (**Figure S3B**). The *uAGL16* mRNA level was slightly (nonsignificantly) lower in response to heat treatment indicating that AGL16 transcription may be affected. To assess the AGL16 promoter activity by a second approach, we created *pAGL16::GUS* reporter lines (**Figure S4A**) and assayed *GUS* mRNA transcription under NT and ACC conditions (**Figure S4B**). The AGL16 promoter activity was decreased in all lines. GUS transcript abundance was mildly induced by *p35S* promoter (*p35S::GUS* was used as a control). These results suggest that the miR824-independent decrease of *AGL16*, at least in part, could be due to a transcriptional downregulation.

Next, we considered the possibility of an elevated decay of *AGL16* mRNA during HS. Cytoplasmic SKI–exosome 3′–5′ exonuclease complex may contribute to the *AGL16* degradation during HS (van Hoof et al., 2002; Halbach et al., 2013). We analyzed the changes of *AGL16* expression in the *ski2-2* mutant (**Figure S3C**). RISC 5′ cleavage fragment of *AGL16* was partially stabilized in *ski2-2* as was shown in the case of other miRNA targets (Branscheid et al., 2015; Szadeczky-Kardoss et al., 2018). The RISC 5′ cleavage fragment stabilization occurred under both NT and HS conditions. These findings suggest that miR824-loaded RISC can operate at elevated temperatures and that the SKI-exosome complex may clear RISC 5′ fragments under both NT and HS. Full-length *AGL16* mRNA decrease was not impaired in *ski2-2* compared to Col-0.

XRN4 is the main cytoplasmic 5′–3′ RNA exonuclease (Souret et al., 2004; Gy et al., 2007; Gregory et al., 2008). XRN4, together with its cofactor LARP1 was shown to play a role in the degradation of unneeded RNA species during early HS (Merret et al., 2013; Merret et al., 2015). We analyzed *AGL16* mRNA dynamics under heat treatment in *xrn4-6* (Gy et al., 2007) and found similar downregulation of full-length *AGL16* as in Col-0 control (**Figure S3C**). Based on these, we failed to attribute any role of SKI–exosome complex or XRN4 in the HS-mediated miR824-independent full-length *AGL16* mRNA decay.

### HS-Induction of miR824 Transcription Requires HSE *cis*-Element

**Figure 4:**
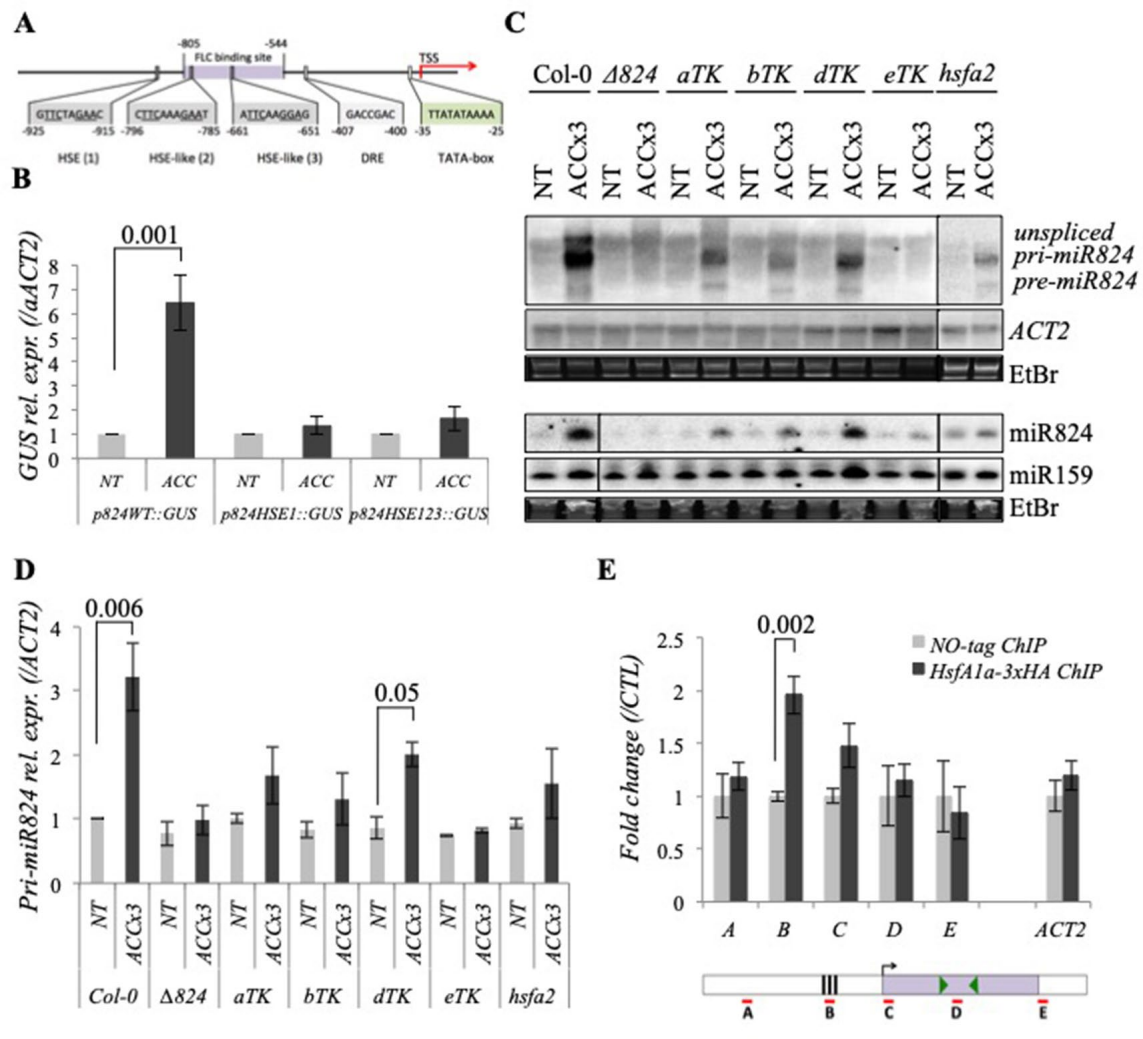
miR824 transcriptional induction requires heat stress elements and heat shock factors. **(A)** Schematic representation of *MIR824* gene promoter: heat stress element, HSE; heat stress element-like motifs, HSE-like; dehydration-responsive element, DRE; TATA-box, FLC-binding site (based on Deng et al., 2011), arrow shows transcription start site, TSS; nucleotide base numbers are relative to TSS. **(B)** Northern blot data of *GUS* expression using the wild-type (*p824_WT_::GUS*), HSE single mutant (*p824_HSE1_::GUS*), and triple mutant (*p824_HSE123_::GUS*) variants of miR824 promoter were quantified to *ACTIN2* and normalized to nontreated control; nontreated, NT, acclimated, ACC; bars represent standard errors calculated based on six representative independent lines each. **(C)** *miR824-precursor* induction and miR824 accumulation are faulty or partial in miR824-mutant *Δ824*, triple mutants of HSFA1 family (*aTK*, *bTK*, *dTK*, and *eTK*) and HSFA2 mutant *hsfa2*. miR159, *ACTIN2*, and ethidium-bromide (EtBr) staining are shown as loading controls. **(D)** Quantification of *pri-miR824* signals of Northern blots; bars represent standard errors based on three biological replicates; Col-0 NT value was set to 1. **(E)** chromatin immunoprecipitation (ChIP) qRT-PCR using 3xHA-tagged HsfA1a expressing transgenic plants: HsfA1a binds to the HSE-containing region of miR824 promoter; data were normalized to no-tag ChIP control; bars represent standard errors calculated based on two biological and two technical reps, schematic representation of *MIR824* locus is shown below: A, B, C, D, E segments show the locations of PCR amplicons; black boxes show the location of HSE elements; green arrowheads show locations of miR824-*5p* and miR824-*3p*; p values based on two-tailed Student's *t*-test.

To better understand the heat-mediated transcriptional regulation of miR824, we studied its promoter *in silico*. We predicted a heat shock element [HSE(1)] at −925 to −915 upstream from the transcriptional start site (TSS) and further two corrupted HSE-like motifs [HSE-like (2) and HSE-like (3)] at −796 to −785 and −661 to −651 upstream from the TSS, respectively (**Figure 4A**). To verify whether these motifs are functional, we employed promoter mutation analysis of our GUS reporter (**Figure 4B** and **Figure S1**). The functionality of the HSE elements was assessed by introducing point mutations to generate a single mutant (*p824* *_HSE1_* *::GUS*) and a triple mutant (*p824* *_HSE123_* *::GUS*) promoter-driven GUS reporter line. Basal and ACC-induced *GUS mRNA* expression of multiple lines was analyzed by Northern blotting and the signals were quantified (**Figure 4B** and **Figure S1B**). Heat induction of the promoter was abolished already when the single HSE element was mutated (**Figure 4A** and **Figure S1**). The expression levels driven from the *p824* *_HSE1_* and *p824* *_HSE123_* promoters were very similar. These results suggest that the HSE motif at −925 to −915 upstream from TSS is functional.

Besides the HSE *cis*-elements, we also found a predicted MADS-box binding site in the promoter of *MIR824* (at −805 to −544 from TSS, **Figure 4A**) using *plantdhs.org* web tool (Zhang et al., 2016). This motif was confirmed as an FLC binding site based on the FLC ChIPseq data of Deng and coworkers (Deng et al., 2011). The presence of the FLC binding motif suggested a possible feedback regulation through an FLC-AGL16 interaction (Hu et al., 2014). To assess the biological relevance of the motif, we checked the miR824 expression in the FLC mutant *flc-2*, wild-type Col-0, and FLC overexpressing Col-*FRI* plants (Michaels and Amasino, 1999) (**Figure S5**). No differences of *pri-miR824* levels could be found between the genotypes under NT or ACC conditions. We cannot exclude however, that FLC with or without AGL16 protein may regulate miR824 expression to fine-tune *AGL16* mRNA levels under more erratic conditions.

### miR824 HS-Induction Requires HSFA1 Family Members and HSFA2 *trans*-Factors

The functional HSE element present in the promoter and transcriptional induction of miR824 during elevated temperatures suggested that heat shock factors (HSFs) might be involved in the process. To determine which HSF is required for *pri-miR824* transcription, we analyzed the miR824 expression in several HSF mutants (**Figures 4C, D** and **Figure S6A**). In the triple knockout mutants expressing only one family member of the HSFA1 family [*aTK*, *bTK*, and *dTK*, (Liu et al., 2011)] the induction of *pri-miR824* was partial while in the triple *hsfa1a;hsfa1b;hsfa1d* (*eTK*) mutant it was completely lost (**Figures 4B, C**). The miR824 HS-induction was partially abolished also in the *hsfa2* mutant (**Figures 4C, D**) but not in the *hsfa3*, *hsfa6a*, *hsfa6b*, *hsfa7a*, and *hsfa7b* mutants (**Figure S6A**). The requirement of HSFA1s and HSFA2 factors for heat-induced miR824 transcription was also confirmed by the decreased accumulation of the mature miR824 in the respective mutant backgrounds (**Figure 4C**). The basal expression of *pri-miR824* or *AGL16* was not significantly altered in the *hsfa1a;hsfa1b;hsfa1d;hsfa1e* (*QK*) (Liu et al., 2011) or in the *hsfa2* mutant (**Figures S6B, C**).

To unravel whether HSFA1s are required for miR824 heat-induction directly or indirectly, we generated transgenic lines expressing 3xHA-tagged HsfA1a from its own promoter in a *QK* background (*QK;pHsfA1a::HSFA1a-3xHA*). The 3xHA-tagged HSFA1a was shown to fully complement the HS phenotype of the *QK* plants (**Figure S7A**) and to be efficiently expressed under HS (**Figure S7B**). We subjected this line to ACC treatment and performed chromatin immunoprecipitation assay (**Figures S7C, D**) followed by real-time quantitative PCR analysis (ChIP-qPCR) (**Figure 4E**). We have found that the genomic DNA located in the HSE-containing promoter region but not the upstream or downstream regions of the *MIR824* locus or *ACTIN2* locus are enriched by HSFA1a-3xHA-ChIP relative to the control sample (**Figure 4E**). These findings are consistent with HSFA1a directly regulating miR824 promoter to activate transcription during heat.

### The *MIR824* Locus Does Not Possess Transcriptional Memory

HS memory-related genes show a faster or stronger transcriptional response upon recurring HS in a process that required the activity of HSFA2 (Lamke et al., 2016). The observation that miR824 transcriptional induction involves HSFA2 activity prompted us to analyze transcriptional memory of the *MIR824* locus. For this, we treated plants repeatedly for one, two or three times (ACC, ACCx2, ACCx3) (**Figure S8**). High and similar levels of *pri-miR824* were detected regardless of the number of acclimations (**Figure S8A**). miR824 induction was neither faster nor stronger, even though a high level of HSFA1a protein was available following the first acclimation (**Figure S8B**). Upon ceasing of heat treatment the *pri-miR824* RNA signal dropped back to background showing that miR824 transcription is neither sustained poststress (**Figure 3D** and **Figure S8A**). We reasoned that the extended ACC program (4 h in total) may saturate the transcriptional induction of *MIR824* gene locus and therefore we may miss the early events. As enhanced transcriptional activation of genes with active transcriptional memory is already apparent after 15 min in response to recurring HS (Liu et al., 2018), we repeated the experiment by applying short treatments (37°C/15 min each). Transcriptional induction of *pri-miR824* was very quick and of similar amplitude regardless of the number of treatments (**Figure S8C**). Based on these we conclude that *MIR824* locus does not possess transcriptional memory.

### The miR824/AGL16 Module Is Not Directly Involved in Heat Stress Response

miR824/AGL16 module was reported to be a regulator of stomata development (Kutter et al., 2007; Yang et al., 2014). Water evaporation through stomata cools the surface of the leaves preventing HS damage. To unravel if *AGL16* downregulation during and following HS has an impact on thermotolerance of photosynthetic apparatus through stomata complexity regulation we measured stomata conductance (gs), CO_2_ assimilation (Pn), transpiration (E) rates, and thermotolerance of the photosynthetic apparatus PS II in NT and ACCx3 plants (Col-0, *agl16-1*, *∆824*, and *MIM824*) at both 25 and 37°C (**Figures S9**–**S11**). No significant changes or consistent trends could be observed between the different genotypes indicating that the temperature-dependent changes of the photosynthetic apparatus are not related to the miR824/AGL16 module (for more details see **Supplementary Information**). In accordance with these, we could not find differences in growth and survival rates of AGL16 or miR824 mutants following basal thermotolerance and short acquired thermotolerance assays [based on (Charng et al., 2007)].

### *AGL16* Tissue-Specific Expression Overlaps with miR824 and FT

**Figure 5:**
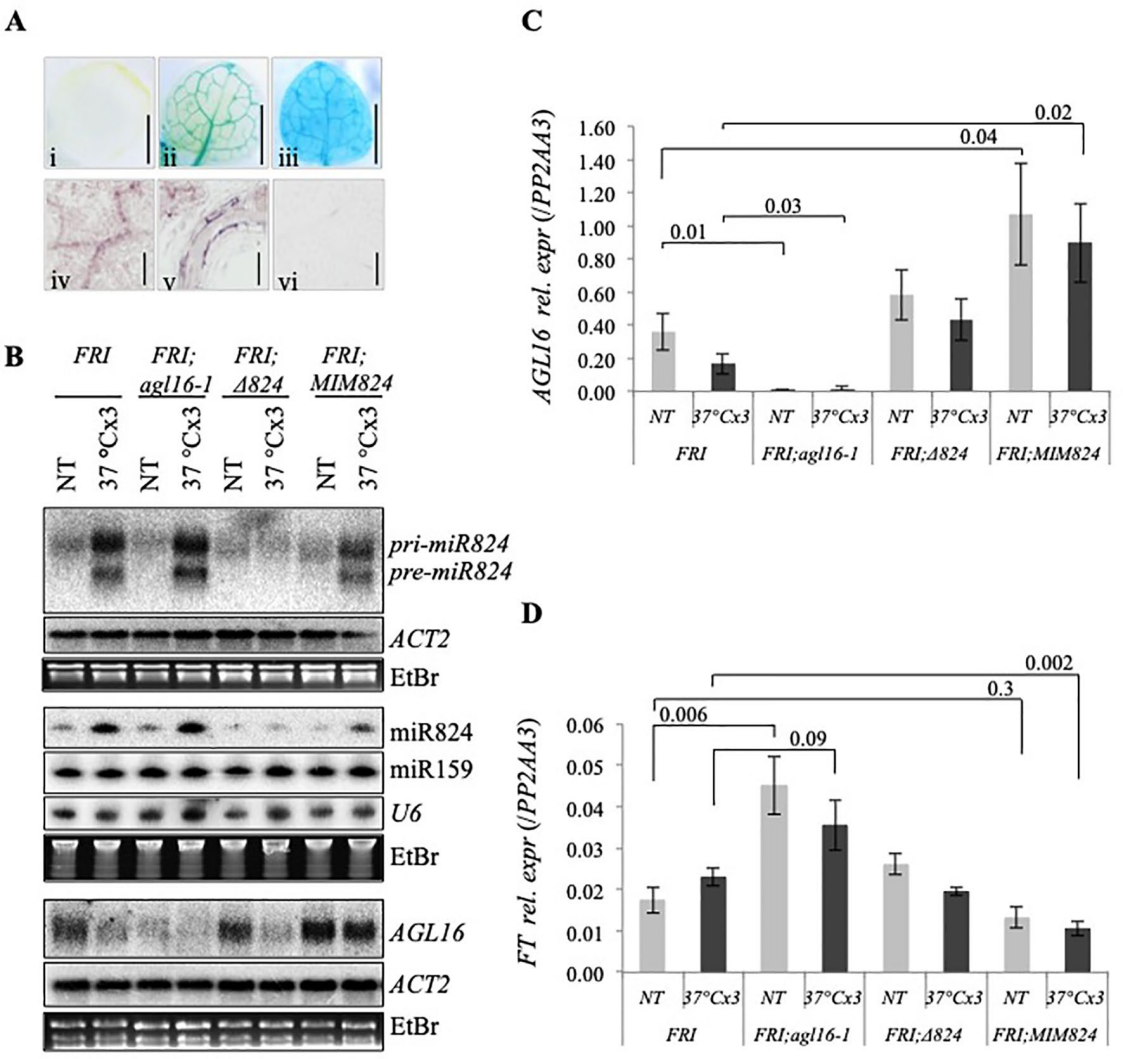
Tissue-specific expression of *AGL16* and expression changes in winter accession *Arabidopsis*. **(A)** *AGL16* expression is specific to vasculature: β-glucuronidase (GUS) activity staining of (i) nontransformed control; (ii) *pAGL16::GUS* transformed; and (iii) *p35S::GUS* transformed seedling leaf (10 day old true leaf, gray bar: 1 mm); *AGL16* messenger RNA (mRNA) *in situ* hybridization of 10 days old seedling: (iv) leaf, (v) root, and (vi) *PIN1* mRNA hybridization control in leaf (gray bar: 0.1 mm); **(B)** *miR824-precursor*, miR824, and *AGL16* mRNA level changes in Col-*FRI* wild-type and mutant plant (samples are shown on the top); *ACTIN2*, miR159, U6, and ethidium-bromide (EtBr) staining are shown as loading controls. **(C)** Heat-induced relative expression changes of *AGL16* in Col-*FRI* wild-type and mutants; bars represent standard errors based on three biological replicates; p values based on two-tailed Student's *t*-test. **(D)** *FT* changes in response to repeated mild HS in wild-type and mutant plants; bars represent standard errors based on six biological replicates; p values based on two-tailed Student's *t*-test.

The other described function of AGL16 is the regulation of flowering transition under light–dark conditions through FT pathway. Manipulation of miR824 abundance in the *MIM824* line contributed to the modulation of *FT* levels and flowering time change (Kobayashi and Weigel, 2007; Hu et al., 2014). Previously it was shown that expression of *FT* and GUS activity (expressed from *pro-miR824::GUS* transgene) localizations are very similar, namely in leaf vasculature (Kobayashi and Weigel, 2007; Hu et al., 2014). However, the tissue-specific localization of *AGL16* was not studied. We analyzed the tissue-specific promoter activity of the AGL16 in transgenic plants expressing GUS transgene from AGL16 promoter (using *pAGL16::GUS* construct) (**Figure 5A**, *i–iii*, and **Figure S4C**). GUS activity was detected in seedling and rosette leaf veins, root calyptra, root cell division, elongation zone, and vasculature, trichomes, shoot apical meristem. We confirmed vasculature localization of *AGL16* mRNA by *in-situ* hybridization (**Figure 5A**, *iv–vi* and **Figure S4D**). The vascular-specific expression of *AGL16* in the leaves overlaps with the expression of both miR824 (**Figure 1D** and **Figure S1A**) and *FT* (Takada and Goto, 2003). These findings extend earlier observations (Burgeff et al., 2002; Hu et al., 2014) and further underpin the role of the miR824/AGL16 module in FT regulation.

### AGL16 Downregulation During and Following Heat Stress May Fine-Tune FT Levels

The impact of the miR824/AGL16 module on FT and flowering acceleration was more pronounced in the background that expressed FLC at high levels (e.g. FRIGIDA) (Hu et al., 2014). We crossed our mutants into the Col-*FRI* background (Col*-FRI*/*agl16-1*, Col*-FRI*/*∆824*, and Col*-FRI*/*MIM824*) and tested the impact of HS on miR824, *AGL16*, and *FT* (**Figures 5B, C**). To avoid the impact of elevated ambient temperature on *FT* (McClung et al., 2016), instead of gradient heat treatments we employed direct 37°C repeatedly (see *Materials and Methods*). This heat treatment enabled efficient induction and accumulation of miR824, and downregulation of *AGL16*; the changes recapitulated the ones found in Col-0 background (**Figures 2A, C**). miR824/AGL16 module heat-regulation, therefore, occurs in both summer (Col-0) and winter (Col-*FRI*) ecotypes of *Arabidopsis*.

To unravel the impact of AGL16 repression on *FT*, we analyzed its mRNA changes in wild type (Col-*FRI*) and mutant (Col-*FRI;agl16-1*, Col-*FRI;∆824*, and Col-*FRI;MIM824*) plants (**Figure 5D**). In Col-*FRI;agl16-1* the *FT* mRNA level was significantly elevated (NT Col-*FRI;agl16-1* vs. NT Col-*FRI*, p = 0.006) with a 2.6-fold difference, similarly as shown before (Hu et al., 2014). Following heat treatment, this difference was partially lost (1.5-fold difference, nonsignificant). In Col-*FRI;∆824* the *FT* mRNA levels were not significantly different from those in Col-*FRI* (in both NT and heat-treated samples) (**Figures 5B, C**). Although only mild changes of *AGL16* are detected in this mutant background the slightly higher *FT* levels (in NT samples) contradicted the expectations (the reason for this is unknown at the moment). In NT Col*-FRI;MIM824* the *FT* mRNA levels were lower compared to NT Col-*FRI* (0.75-fold difference, nonsignificant). This is in agreement with the finding that *AGL16* levels are high in the absence of miR824 activity (**Figure 5C**). *FT* levels dropped significantly following heat treatment in Col*-FRI;MIM824* (0.46-fold difference, p = 0.002, **Figure 5D**). As *FT* changes were mild we wanted to corroborate these findings: we analyzed *FT* changes using another internal control (*UBC22* mRNA) and got similar results (**Figure S12**).

These results suggest that HS has a complex impact on *FT*, probably through multiple pathways, including AGL16-independent and AGL16-dependent ones. The heat-induced downregulation of *AGL16* (in wild-type) may cause a mild derepression of *FT*. In the absence of *AGL16* changes (e.g. Col*-FRI;agl16-1* or miR824 mutants), the level of *FT* slightly drops, suggesting that HS may impact it negatively through AGL16-independent pathways. miR824/AGL16 module, therefore, may compensate for the retarding impact of heat under mild HS conditions (in wild type), while in *agl16-1* and miR824 mutants, where this buffer system is not available, the negative impact of HS on *FT* becomes apparent (see also *Discussion*). Prompted by the observation on *FT* changes, we tried to assay the impact of HS on the timing of the flowering transition. Unfortunately, we could not detect consistent differences in flowering time following our heat treatments between mutants and wild type plants (see also *Discussion*).

### miR824/AGL16 HS-Regulation Is Conserved Within *Brassicaceae*

**Figure 6:**
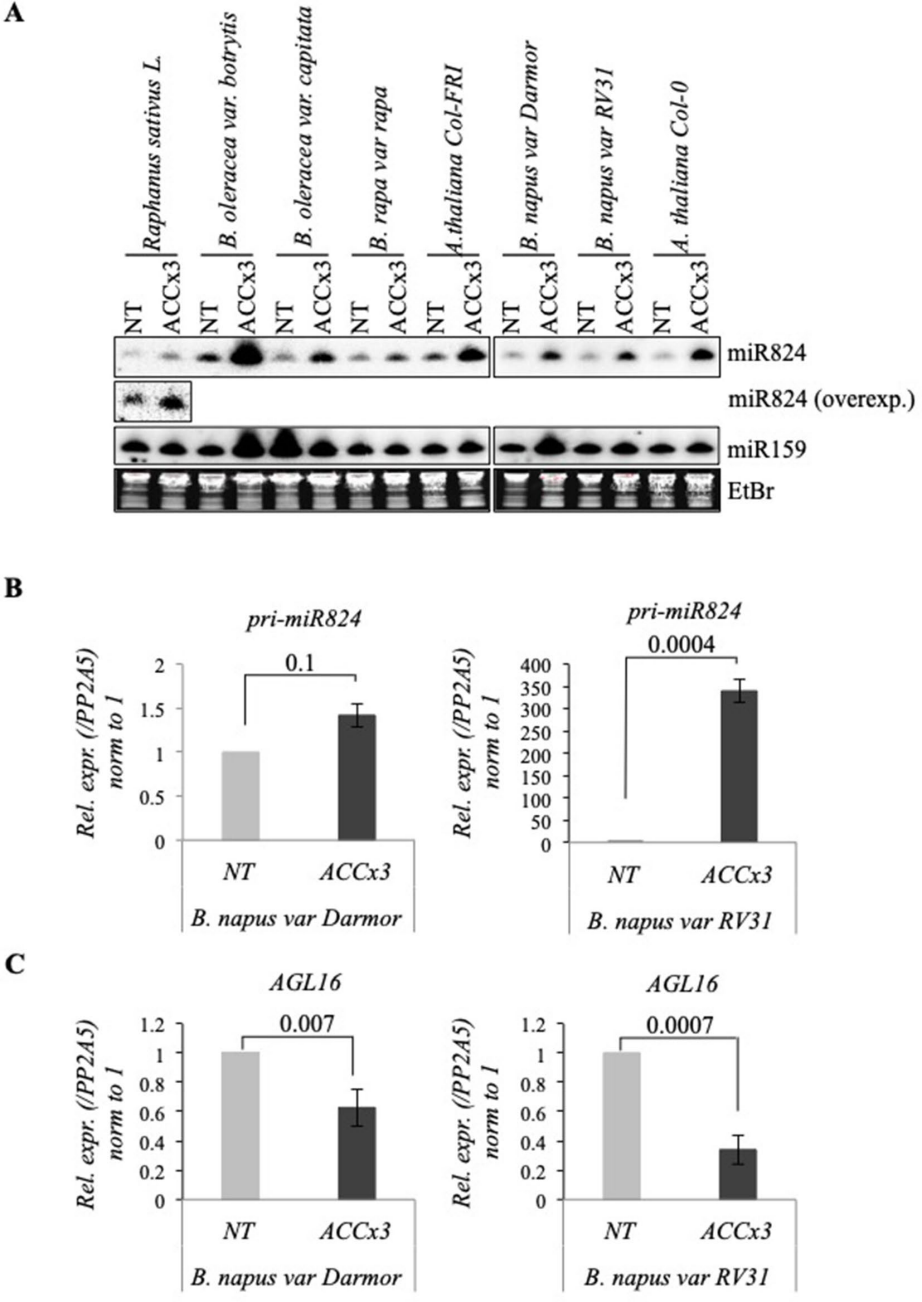
Heat-regulation of miR824 and *AGL16* is conserved in *Brassica* species. **(A)** miR824 accumulates in *Brassicaceae* family members in response to recurring acclimation. miR159 and ethidium-bromide (EtBr) staining are shown as loading controls. **(B)** *pri-miR824* changes in *B. napus* winter cultivar *Darmor* and the spring cultivar *RV31*. **(C)** *BnaAGL16* is significantly depleted during repeated acclimation in *B. napus*; NT value was set to 1. bars represent standard errors based on three biological replicates; p values based on two-tailed Student's *t*-test.

Both miR824 and AGL16 (containing miR824-RISC target site) are conserved within *Brassicaceae* (Kutter et al., 2007; de Meaux et al., 2008). We performed a complementary experiment to check whether HS-regulation of miR824/AGL16 functional module is conserved. First, we assayed miR824 behavior in response to ACCx3 in multiple members of *Brassicaceae* (*B. rapa*, *Brassica oleracea*, *Brassica napus*, and *R. sativus*). miR824 accumulated in all *Brassica* plants tested (**Figure 6A**). To confirm that miR824 accumulation is due to transcriptional induction, we checked miR824 precursors in *B. napus* winter variety *Darmor* (containing active FRI paralogs) and the summer variety *RV31* (*Westar* derivative) by qRT-PCR: *pri-miR824* was elevated following heat acclimation in both varieties (**Figure 6B**). In parallel to this, *AGL16* mRNA downregulation also occurred in the two *B. napus* varieties (**Figure 6C**). Altogether these observations suggest that HS-regulation of miR824/AGL16 module is conserved within *Brassicaceae*, and may have a role in fine-tuning adaptation following mild and repeated HS.

## Discussion

Plants respond to diurnal and seasonal changes in temperature by reprogramming their developmental pathways. It is known that the impact of HS largely depends on the strength, type, and duration of stress or the developmental stage of the plants when stress is encountered (Yeh et al., 2012). While the effect of direct HS on miRNA regulation has been intensively studied (Ballen-Taborda et al., 2013; Guan et al., 2013; Cui et al., 2014; Kruszka et al., 2014; Stief et al., 2014; Liu et al., 2015) much less is known about how gradual and repeated high temperatures affect miRNAs. Our HS program was designed to mimic natural conditions involving both the gradual and repeated aspects. Regimes involving gradual stress buildup are physiologically relevant since they resemble natural situations. Recurring high or low temperatures, gradual onset of drought and salt may all lead to the accumulation of stress-responsive miRNAs.

Using this program we have found that miR824 is a HS responsive miRNA. We have characterized in detail the changes of the miR824/AGL16 module in response to heat and in the poststress period. Using an *in silico* analysis and GUS reporter assay we demonstrated that MIR824 gene promoter contains a functional HSE cis element. We also propose that HSFA1a *trans* factor may directly bind to the HSE containing region of MIR824 promoter (**Figure 4E**). Besides HSE, we have also found a drought-responsive element in the promoter of MIR824 (GACCGAC, −407 to −400 from TSS) (**Figure 4A**). The combination of heat and drought induced strong transcription of *pri-miR824* and accumulation of miR824 (**Figure S13**). Beyond these, miR824 was shown to be downregulated by arsenic stress in *B. juncea* (Srinivasan et al., 2006) and to accumulate under chromium stress in *R. sativus L.* (Liu et al., 2015). On the other hand, AGL16 homolog genes in *B. rapa*, *BraMADS20* and *BraMADS21* showed differential accumulation in response to cold or salt stress (Saha et al., 2015). Our findings and data from the literature suggest that miR824/AGL16 pathway may integrate the stimuli of multiple abiotic stresses under complex climatic conditions. It remains a future task to understand the role of miR824/AGL16 module under combined stresses.

We show that although miR824 transcription is transient, mature miR824 accumulates gradually to high levels following repeated heat treatments (**Figure 1C**). Similarly, stress-induced changes of miR168 and miR171a-1 were found by using repeated HS regime (42°C/3 h per day/7 days in a row) (Bilichak et al., 2015; Liu et al., 2015). As miRNAs possess long half-life (Csorba et al., 2010; Gantier et al., 2011; Sanei and Chen, 2015) they may be capable to act as integrators of stress signals over a few days. The exploitation of heat spikes through miR824/AGL16 module changes may serve plants for monitoring the seasonal progression, similarly as shown before in another system (Hepworth et al., 2018).

**Figure 7:**
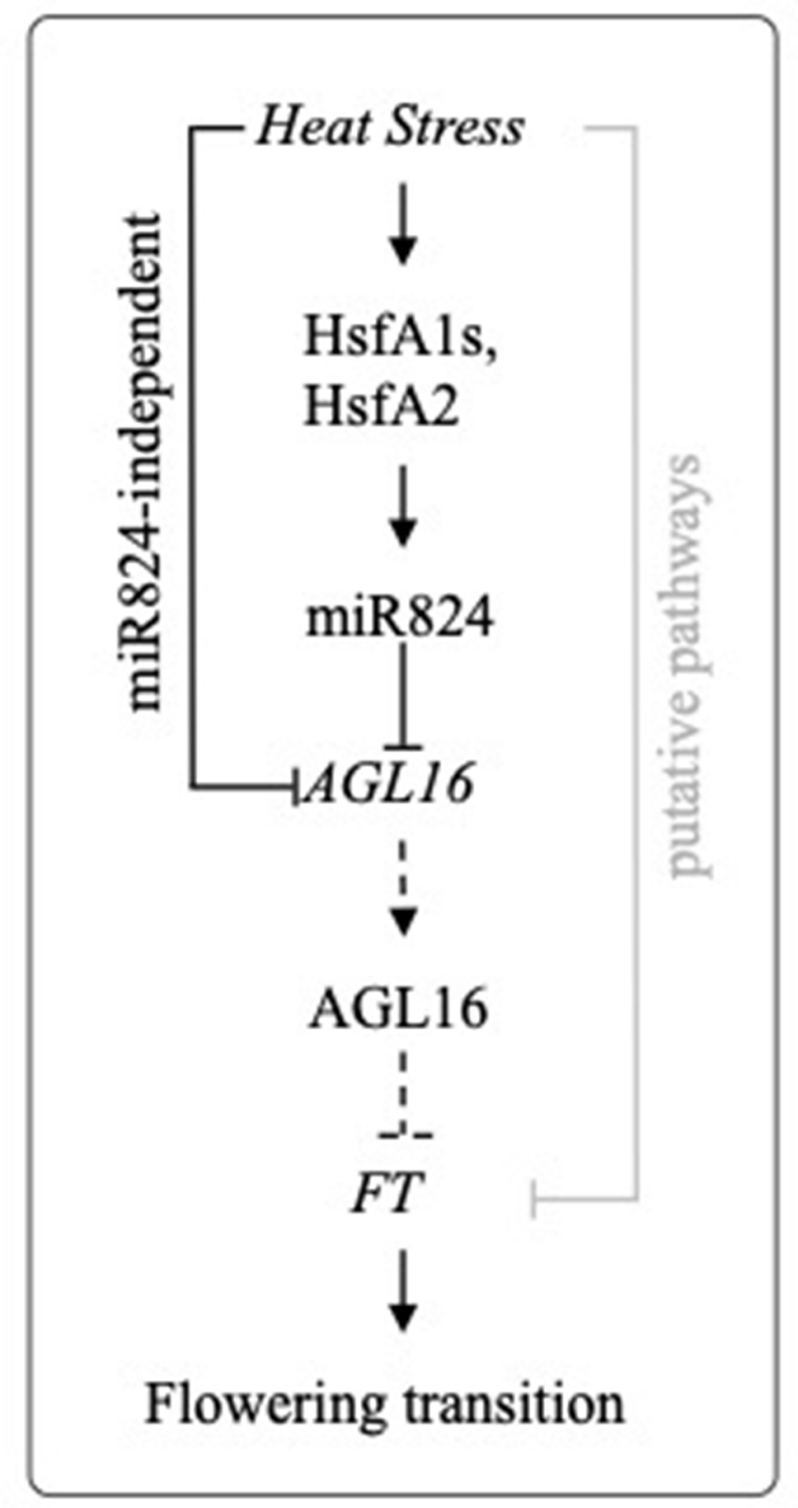
Proposed working model of miR824/AGL16 module heat stress regulation. Heat stress induces transcription of miR824 through HSFA1a family and HSFA2 transcription factors. *AGL16* is depleted through a miR824-dependent and a miR824-independent pathway. Stable downregulation of *AGL16* leads to derepression of *FT*, a central integrator of flowering transition (dotted lines depict downregulated steps during and following HS). *FT* level may be also altered by other putative heat stress (HS)-regulated factors (gray line).

We studied miR824 unique target AGL16 and have shown that its downregulation in response to high temperatures is dual, comprising of a miR824-independent and a miR824-dependent route (see *Working Model*, **Figure 7**). *AGL16* nascent transcript level and promoter activity are decreased miR824-independently during heat (**Figures S3B** and **S4B**), therefore transcriptional ceasing contributes to *AGL16* downregulation. As reduction is abrupt, mRNA decay may be also involved (**Figure S3C**). HS-mediated decay was shown to be an important component of HSR (Merret et al., 2013; Merret et al., 2015). To confirm our findings, we have analyzed the transcriptome changes in *larp1* RNAseq data (Merret et al., 2013) and *xrn4-5* RNAseq data (Merret et al., 2015): *AGL16* transcript was enriched in neither *larp1* nor *xrn4-5* vs. wild-type as it would be expected if XRN4-LARP1 pathway is involved in its degradation. HS-induced *AGL16* transcript changes found by (Merret et al., 2015) are remarkably similar to the ones found by us: *AGL16* is downregulated to 0.62 in wild-type Col-0 and 0.69 in *xrn4-5* following 30 min of HS. It is possible that 3′–5′ XRN4 and 5′–3′ SKI–exosome pathway act redundantly to contribute to *AGL16* mRNA decay. miRNA-independent heat-induced downregulation of miRNA targets was observed in other cases as well: *ARF10, 16* and *17* targets of miR160 were partially and significantly downregulated by heat even when the effect of miR160 was blocked through the expression of mimicry constructs (Lin et al., 2018).

Several lines of evidence support the involvement of miR824-dependent pathway in downregulation of *AGL16*: (i) basal levels of *AGL16* in wild-type (both Col-0 and Col-*FRI*) is lower compared to the miR824-pathway deficient *∆824* and *MIM824* lines; consistent with this, *AGL16* level is elevated in *dcl1-8*, *hyl1-1*, and *hen1-1* mutants (Kutter et al., 2007; Confraria et al., 2013) (ii) repression of *AGL16* following heat treatment persists for several days only in wild-type plants but not miR824-deficient mutants; (iii) RISC 5′ cleavage fragments in *ski2-2* plants become stabilized in both NT and HS samples. This latter observation also suggests that RNA silencing is active during HS.

Importantly, we show that the extended presence of miR824-loaded RISC prolongs the effect of heat and maintains the low levels of *AGL16* target poststress (**Figure 3**). *miR156* family members are also at elevated level poststress and this results in stable downregulation of target *SQUAMOSA-PROMOTER BINDING-LIKE* (*SPL*) transcription factors mRNA (Stief et al., 2014). As suggested by the authors, the lasting high level of miR156 mature form is probably due to the combination of transcriptional memory of the locus and the high miRNA stability (Stief et al., 2014). In the case of miR824 we excluded transcriptional memory of the locus (**Figure S8**). Transient HS-inducibility (absence of sustained transcription poststress) of miR824, therefore, provided us an excellent tool to measure its half-life and track its downstream effects (instead of using general transcription inhibitors like cordycepin that have a strong pleiotropic impact). Following a single heat treatment, during which the miR824 transcription is transiently switched on, we have shown that the mature miRNA persists and is active for several days (**Figure 3**). In addition to these, we analyzed the heat-inducible miR398a (Guan et al., 2013) and found elevated levels of it 3–4 days poststress (**Figure S14**). As stress-induced miRNAs are stably present poststress, as shown for miR824, miR398a (in this study) or miR156, miR831 (Stief et al., 2014), and active in repressing their targets, we propose they should be regarded as posttranscriptional stress-memory factors. The lasting effect of stress-induced miRNAs enables plants to "remember" the recent occurrence of stress and helps to alter the poststress development on a few days timescale or during intermittent periods between stresses. Whether stress-induced sRNAs act as memory factors during other or combined stress conditions remain a future and exciting question.

The timing of flowering is a critical trait that ensures the perfect timing of seed production required for species survival. Transition to flowering is regulated by an elaborate network involving numerous players based on endogenous and exogenous stimuli. Age, circadian clock, sugar content, temperature, and hormonal pathways converge on a limited number of master regulators (Romera-Branchat et al., 2014; Whittaker and Dean, 2017). Temperature is one of the most important environmental stimuli to modulate transition timing from vegetative to reproductive phase. It is known that elevated ambient temperature accelerates flowering time in *Arabidopsis* (Balasubramanian and Weigel, 2006; Capovilla et al., 2015; McClung et al., 2016). How nonlethal HS affects flowering time is much less understood.

Our data suggest that *AGL16* downregulation during and following HS may contribute to a mild derepression of *FT*. In wild-type plants, *FT* is slightly elevated, while in AGL16/miR824 mutants is rather repressed. We hypothesize that the AGL16/miR824 module may serve as a buffer system to dampen the effects of HS that retards the flowering transition (**Figure 7**). Unfortunately, we could not detect flowering time changes following our HS program. Several factors could hinder this. As the heat treatment is mild and plants are exposed for a relatively short period, our treatment may have a limited impact/potential to cause lasting changes that culminate in flowering. Proper timing of HS is also critical and difficult to predict. Furthermore, FT-independent pathways (Wang et al., 2009) also alter flowering time. One such pathway studied in detail is the miR156/SPL module pathway (Stief et al., 2014). SPLs are master regulators of developmental transitions and accelerate flowering in an FT-independent manner (Wang et al., 2009). HS-induced miR156 negatively regulates *SPLs* (Stief et al., 2014). miR156/SPL pathway coordinates the balance between development and stress response in the favor of the latter, to delay flowering (Cui et al., 2014; Stief et al., 2014) therefore act in the opposite direction (compared to the impact of miR824/AGL16). Recently it was shown that *Arabidopsis* plants exposed to prolonged mild HS temperatures (30°C for 7 days) bolted earlier and that early flowering phenotype was transmitted trans-generationally for two generations (Liu et al., 2019). Early flowering and transgenerational thermomemory were caused by a complex regulatory network that culminates in the release of *HEAT-INDUCED TAS1 TARGET 5*. *HEAT-INDUCED TAS1 TARGET 5* drove early flowering in a process involving FT upregulation (Liu et al., 2019). In summary, better understanding the interaction between positive and negative regulators and combined impact on developmental transitions following nonlethal HS conditions remains a future and exciting challenge.

FT and FLC orthologs are key genes that contribute to flowering time control and a successful adaptation to diverse environmental conditions and geographical distribution in the different *B. napus* ecotypes (Wu et al., 2019). We show that HS regulation of the miR824/AGL16 module is conserved in several *Brassica* species and characteristics to both summer and winter varieties (**Figure 6**). The paralog *rsa*-miR824/AGL16 module in radish was also related to bolting and flowering processes (Nie et al., 2015). HS regulation of the miR824/AGL16 module may, therefore, help successful adaptation of *Brassica* species and fine-tune the trade-off between stress and development.

## Data Availability Statement

Raw RNAseq data have been made available in the SRA repository (SRP151884).

## Author Contributions

TC conceived the original research plans and designed the experiments. HMS and TC performed most of the experiments and analyzed the data. ÉD and TJ performed physiological measurements. H-CL and Y-YC prepared the *phsfa1a::HsfA1-3xHA*-tagged transgenic plant lines and provided technical assistance. AM performed the *in situ* hybridizations. TC wrote the article with contributions of all the authors.

## Funding

TC was supported by the János Bolyai Research Scholarship of Hungarian Academy of Science. Y-YC was supported by a grant from the Ministry of Science and Technology, Taiwan (103-2311-B-001-011-MY3). Hungarian Scientific Research Fund OTKA K115934, K129283 funded this work.

## Conflict of Interest

The authors declare that the research was conducted in the absence of any commercial or financial relationships that could be construed as a potential conflict of interest.
