# Supplementary material for: miR824/AGAMOUS-LIKE16 Module Integrates Recurring Environmental Heat Stress Changes to Fine-Tune Poststress Development
Source: Front Plant Sci. 2019 Nov 25;10:1454. doi: 10.3389/fpls.2019.01454 (PMC6886564; doi:10.3389/fpls.2019.01454)
Supplement: Supplementary file 1 [file DataSheet_1.pdf]

Supplementary information

HS-regulation of miR824/AGL16 module does not affect stomata conductance and photosynthetic activity

miR824/AGL16 module was reported to be a regulator of stomata development (Kutter et al., 2007; Yang et al., 2014). Stomata are vital structures that are required for water and carbon dioxide uptake during photosynthesis. Water evaporation through stomata cools the surface of the leaves, preventing heat-stress damage of membranes and proteins. To unravel if *AGL16* down-regulation during and following heat stress has an impact on thermo-tolerance of photosynthetic apparatus through stomata complexity regulation we measured stomatal conductance (gs), CO_2 assimilation (Pn) and transpiration (E) rates of NT and ACCx3 plants (Col-0, *agl16-1*, Δ*824* and *MIM824*) at both 25 °C and 37 °C (Fig S9). In addition, the thermo-tolerance of the photosynthetic apparatus PS II was also investigated (Fig S9). The CO_2 assimilation activity (Pn) was lower while stomatal conductance (gs) and transpiration rate (E) were higher at 37 °C than at 25 °C (Fig S9) in leaves of both NT and ACCx3 plants. Acclimation treatment (NT vs. ACCx3 plants) modified the gas exchange parameters (Pn, gs, and E) and enhanced the thermo-tolerance of PSII as demonstrated by the temperature-dependent changes of effective quantum yield of PS II parameter (Y(II))(Fig S9A and S10). Genotypic variations were found for gs and Y(II), but no significant changes could be observed in Pn and E parameters between the different genotype groups. Since these changes did not show consistent trends, the results can't prove evidently that the temperature-dependent changes of the photosynthetic function are related to the *miR824*/AGL16 module (Fig S11). In accordance with these, we could not find differences in survival rate following different HS regimes. All these results suggest that *miR824*/AGL16 module might affect indirectly the thermo-tolerance.

Validation of agl16-1 mutant by RNA-seq

We detected an *AGL16* signal in *agl16-1* by the northern blot analysis (Fig 2A and Fig 5B). To make sure this is a specific signal, we confirmed *agl16-1* mutation by genotyping (Fig S2A), by testing the levels of *AGL16* in a qRT-PCR reaction (primers are spanning on the two sides of the SALK insertion)(Fig 2C and 5C) and a physiological assay (changes in FT, Fig 5D). *agl16-1* T-DNA insertion is located in

the last exon of *AGL16* (Fig S2B). We reasoned that a combined *AGL16*-T-DNA fused transcript is detected during northern hybridizations. Of note, *AGL16* signal in *agl16-1* is also depleted during heat similarly to the *bona fide AGL16* in wild-type Col-0 (Fig 2). To verify this possibility we analyzed RNA transcriptome data of *agl16-1* mutant and Col-0 wild-type plants (Fig S2B)(SRP151884). Comparable (slightly lower) read numbers were mapped along the *AGL16* exons in *agl16-1* (compared to Col-0 control) except for the last exon: here, at the place of T-DNA insertion, read numbers abruptly dropped. This confirms that the *AGL16* northern blot signal in *agl16-1* is specific (Fig 2 and 5). This *AGL16-T-DNA* transcript has a lower abundance and may translate a non-functional or at least hypo-functional protein.

Primer Table

Geno-typing	*lb1_sail*	gcc ttt tca gaa atg gat aaa tag cct tgc ttc c	
	lbb1_salk	gcg tgg acc gct tgc tgc aac t	
	p745_wisc	aac gtc cgc aat gtg tta tta agt tgt c	
	hsfa1a-5	aag aag ata agc cgg aga aaa tct	
	hsfa1a-6	aca aag ttg caa ccg tac tac tga	
	hsfa1b-5	cca gct tcg tca gac agt taa ata	
	hsfa1b-6	tag gaa act gtc agg att gtt tga	
	hsfa1d-5	gca taa taa ttt ctc cag ctt cgt	
	hsfa1d-6	agg ttt tcg cct agt tat tga ttg	
	hsfa1e-5	ttt taa gag gcc aaa agc aaa tac	
	hsfa1e-6	gtt gat tct tgc tcc aca cat tac	
	hsfa2_glp	aaggttccgaaccaagaaaac	
	hsfa2_grp	ctcaacaactctccttccacg	
	hsfa3_glp	aaa aga taa atc cac ggt ggc	
	hsfa3_grp	agc aag ttt ggt tgg att gtg	
	hsfa6a_glp	tca ctc aac acg aaa ccc ttc	
	hsfa6a_grp	ttc act aca acg tgt cat ggg	
	hsfa6b_glp	gtt ttg tcc gcc agc tca aca c	
	hsfa6b_grp	cct tag gct gtc cat ctc tcc	
	hsfa7a_glp	tgg agg gtt tac acg aaa atg	
	hsfa7a_grp	agc cag aaa cga cat cat ttg	
	hsfa7b_glp	ttc ttc gca agt tct gga aac	
	hsfa7b_grp	tcc cat ttt ata aga ttt tca agc	
	agl16-1_glp	acc tcc aca aga aag taa acc taa tgc	
	agl16-1_grp	cgg ttg gct gag ctg aag at	

	xrn4-6_glp	aggtgtatgctcttggcaatg	
	xrn4-6_grp	aactgccatgaaaactgatgg	
	ski2-2_glp	actcggaatcgttctgaagg	
	ski2-2_grp	tcccatcctcgagtaggcgc	
	fri_glp	ttgataaggatgagtggttcga	
	fri_grp	tgtcaacaaaaggaaccacctt	
Cloning	*prom824_F-2841*	atatgaattcacggtctgatgcgatgatcc	
	prom824_R102	atatccatgg<br>gtcggaaaaagccgtgatgtg	
	p824HSE1m_F	gtattatcaaatcttttgtgctagcacctc	
	p824HSE1m_R	tatttaaagtttaagaggtgctagcacaaa	
	p824HSE2m_F	cgtgtggtccttaaaataataaccagcg	
	p824HSE2m_R	agatttcgctggttattattttaaggac	
	p824HSE3m_F	catttgtattcattaaatgagacaagta	
	p824HSE3m_R	taatttacttgtctcatttaatgaatac	
	proAGL16_F1gap	taacaatttcacacaggaaacagctatgacc<br>atgattacgtgaaaccctgttaatctttt	
	proAGL16_R1gap	aaatgaaggagaaaaactagaaatttaccct<br>cagatctacctgaatttctgaagcgggat	
Northern blot probe generation	*ACT2_F79*	ggctggatttgcaggagatg	
	ACT2_R823	cttgcccatcgggtaattca	
	AGL16_F742	ccg gta ggc tct acg att tct	AGL16_R3489 for Northern probe
	AGL16_R3489	cgg ttg gct gag ctg aag at	
	AGL16_F_3'FR	cgaacatgtccatcttcagc	
	AGL16_R_3'FR	aaattgatttggatgggaagc	
	PRIMIR824_F147	tcc gcc att ttc gaa att ctt	(combine with miR824-5p for genotyping, combine with miR824-3p for northern probe generation)
	MIR824A-5P	tcc ctt ctc aca aat ggt cta	
	MIR824A-3P	tct aga cca tcg atg aga agg	
	MIR159	tagagctcccttcaatccaaa	
	U6	*gctaatcttctctgtatcgttcc*	
	CSD1_F	*atggcgaaaggagttgcagttt*	
	CSD1_R	*ttagccctggagaccaatgatgc*	
	MIR398a	*tgtgttcacatgccactcctt*	
	MIR156h	gtgctctctttcttctgtca	
qrtPCR	ACT2-Fqrt	cgc tct ttc ttt cca agc tca t	
	ACT2-Rqrt	gca aat cca gcc ttc acc at	
	FLC_Fqrt	agccaagaagaccgaactca	
	FLC_Rqrt	tttgtccagcaggtgacatc	
	FT_Fqrt	ggt gga gaa gac ctc agg aac t	
	FT_Rqrt	ggt tgc tag gac ttg gaa cat c	
	BnaPP2A5_Fqrt	atctcttcatgggcgattacgttga	

	BnaPP2A5_Rqrt	agcgaactttgagtgctaccaag	
	Bna-pri824_Fqrt	ggtaaagagaaatggtgatttatagga	
	Bna-pri824_Rqrt	caaaacaataattccaaaagctga	
	AGL16_F2630	acc tcc aca aga aag taa acc taa tgc	
	uAGL16_R	cgacctctgaaacctggaat	(combine with F2630 for unspliced)
	AGL16_R3489	cggttggctgagctgaagat	(combine with F2630 for spliceed)
	PP2A_F	tcttttcatgggtgattatgttga	
	PP2A_R	aacgaactttcagtgctaccaaa	
	BnaAGL16_qF	gcaggctctacgagttctcc	
	BnaAGL16_qR	tttggcctcgctgtatctatc	
	PP2AA3_F	cctgcggtaataactgcatct	
	PP2AA3_R	cttcacttagctccaccaagca	
	RD29A_F	agg aac cac cac tca aca ca	
	RD29A_R	atc ttg ctc atg ctc att gc	
	UBC22_F	tcctcttaactgcgactcagg	
	UBC22_R	gcgaggcgtgtatacatttg	
ChIP	p824_-2841F_chipA	acggtctgatgcgatgatcc	
	p824_-2699R_chipA	ttcaaaacgtggcagggaac	
	p824_-978F_chipB	agttcgactcaactacactgctttaat	
	p824_-828R_chipB	ttttggctcgtccagtttttg	
	p824_25F_chipC	ccctctctcgcatccttcct	
	p824_105R_chipC	acagtcggaaaaagccgtga	
	p824_902F_chipD	ggtaaaaaggagctcgtggaaa	
	p824_986R_chipD	ttgccgaagaagaagaacgaa	
	p824_2891F_chipE	gagtttaaattgcatgcgtataaaaga	
	p824_986R_chipE	tcggatgcacccaaccatta	
	ACT2_chipF	tgccaatctacgagggtttc	
	ACT2_chipR	tctcttacaatttcccgctctg	

Supplementary references

Kutter C, Schob H, Stadler M, Meins F, Si-Ammour A (2007) MicroRNA-mediated regulation of stomatal development in Arabidopsis. Plant Cell **19:** 2417-2429

Yang K, Jiang M, Le J (2014) A new loss-of-function allele 28y reveals a role of ARGONAUTE1 in limiting asymmetric division of stomatal lineage ground cell. J Integr Plant Biol **56:** 539-549
