# Supplementary figures and images for: miR824/AGAMOUS-LIKE16 Module Integrates Recurring Environmental Heat Stress Changes to Fine-Tune Poststress Development
Source: Front Plant Sci. 2019 Nov 25;10:1454. doi: 10.3389/fpls.2019.01454 (PMC6886564; doi:10.3389/fpls.2019.01454)

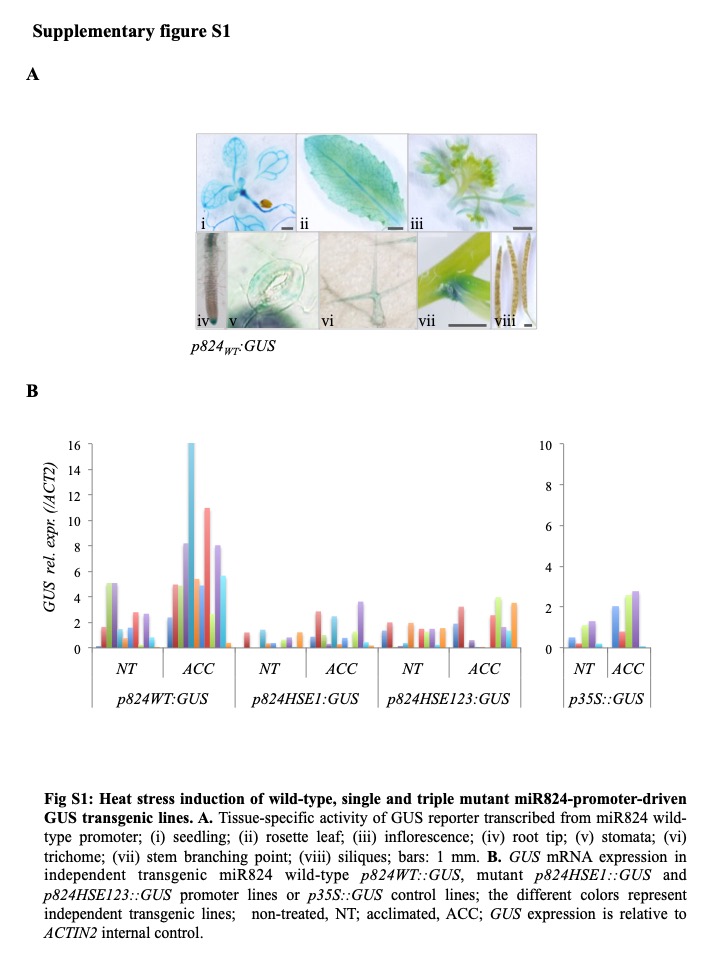

Supplement: Supplementary file 2 [file Image_1.jpeg]

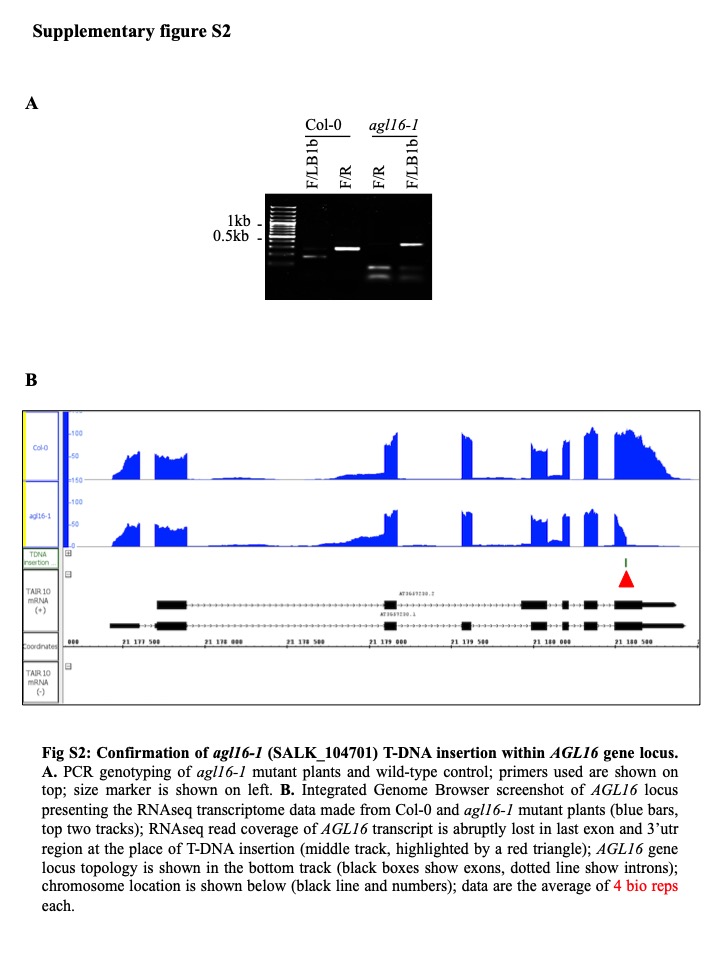

Supplement: Supplementary file 3 [file Image_2.jpeg]

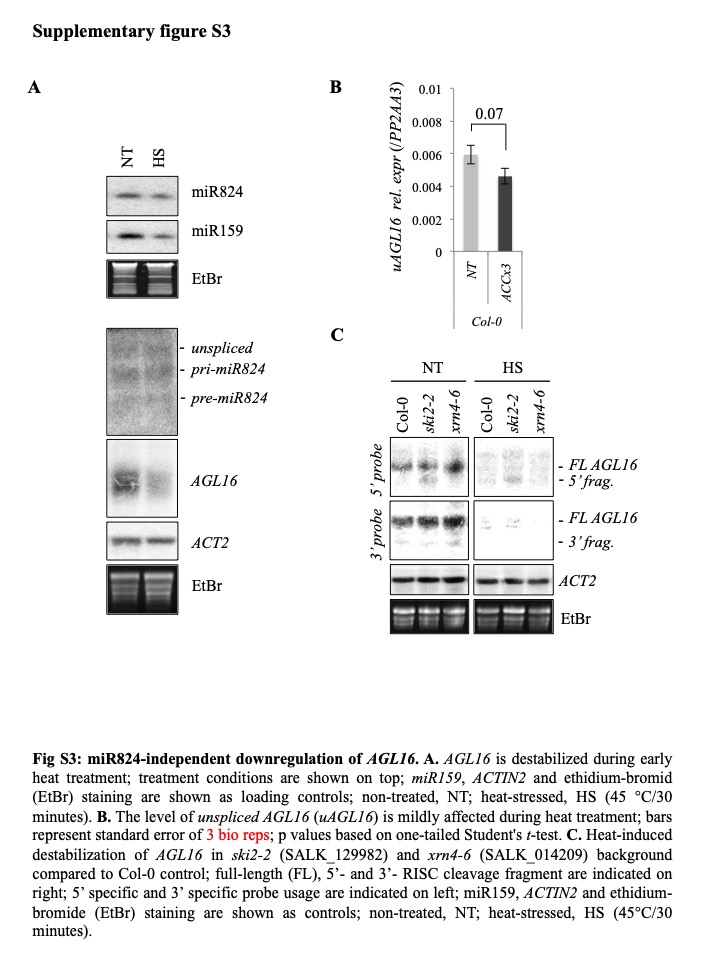

Supplement: Supplementary file 4 [file Image_3.jpeg]

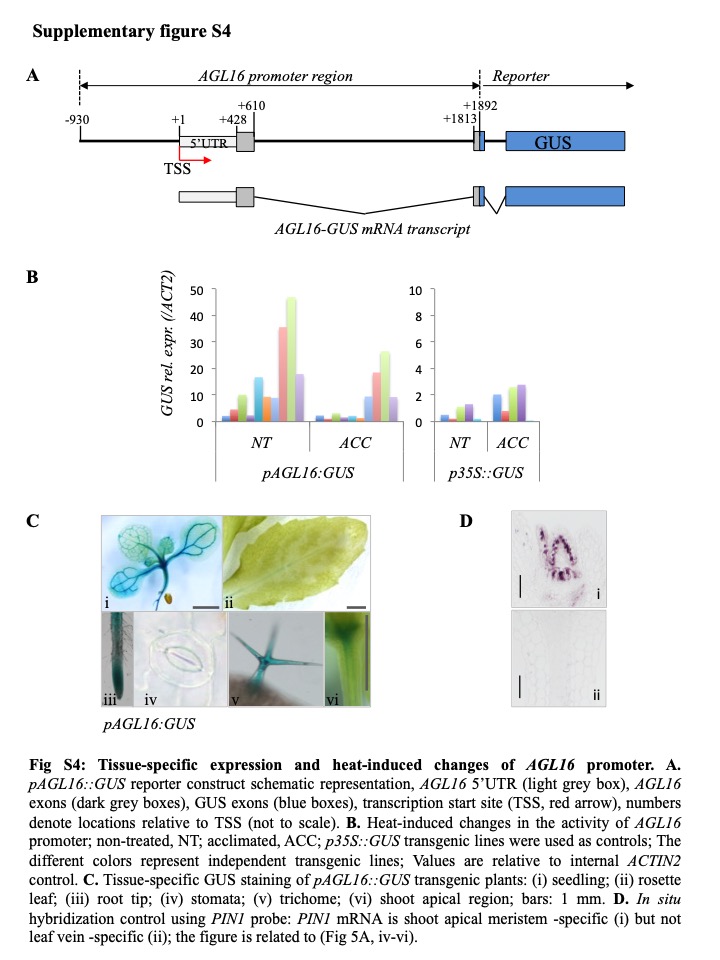

Supplement: Supplementary file 5 [file Image_4.jpeg]

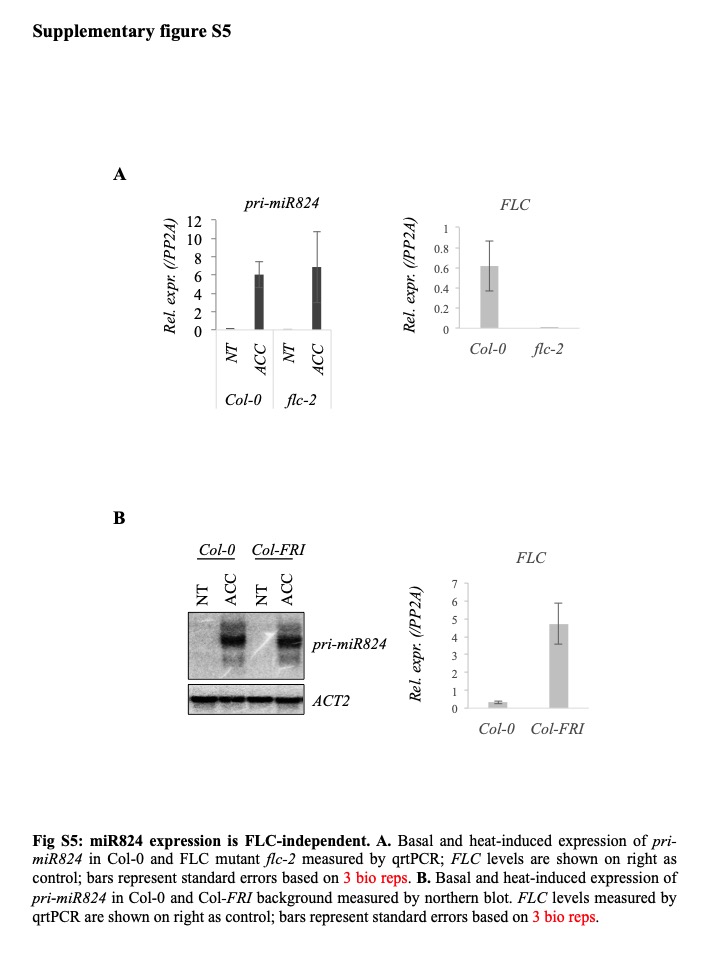

Supplement: Supplementary file 6 [file Image_5.jpeg]

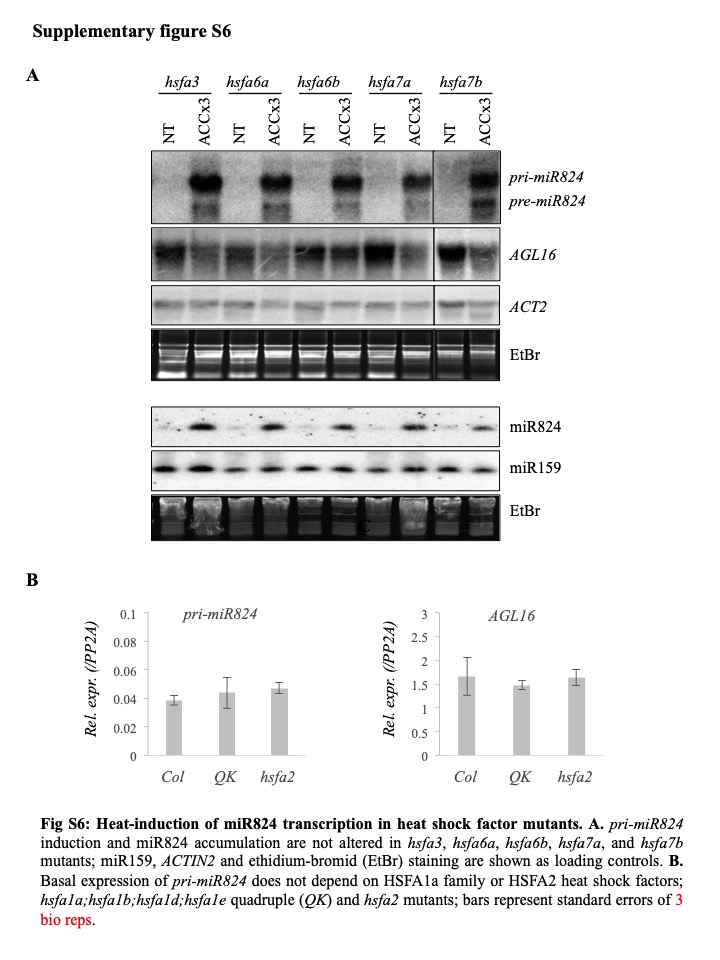

Supplement: Supplementary file 7 [file Image_6.jpeg]

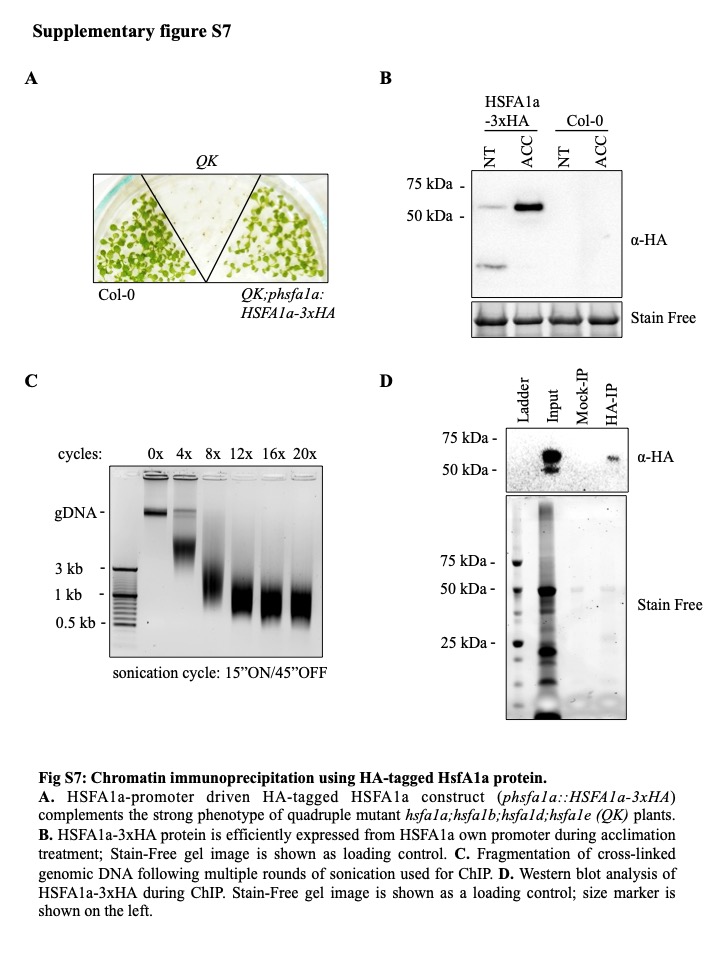

Supplement: Supplementary file 8 [file Image_7.jpeg]

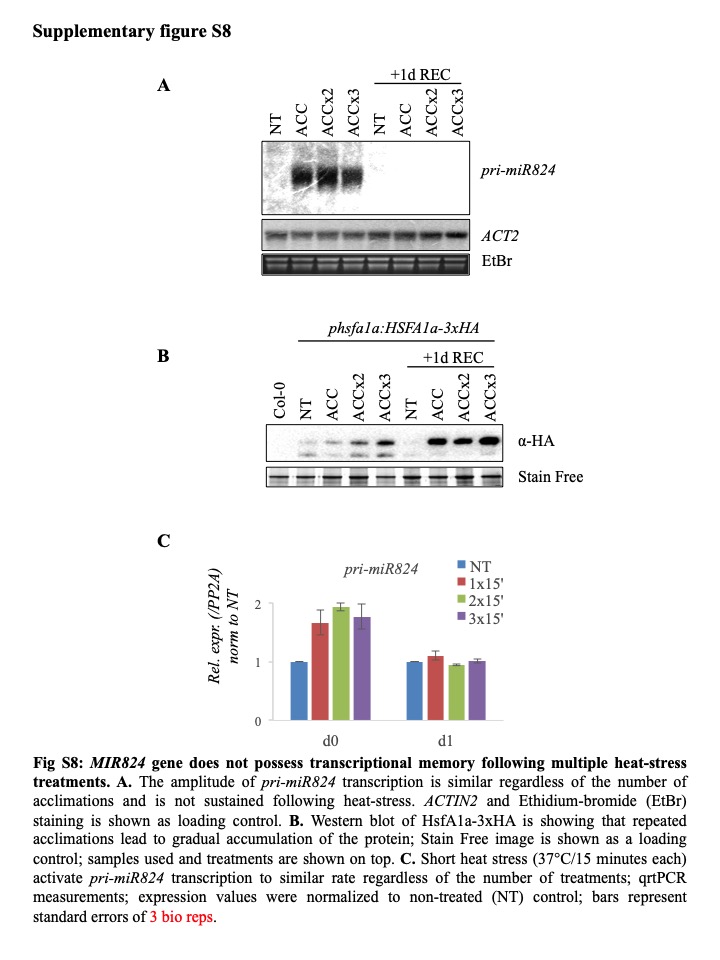

Supplement: Supplementary file 9 [file Image_8.jpeg]

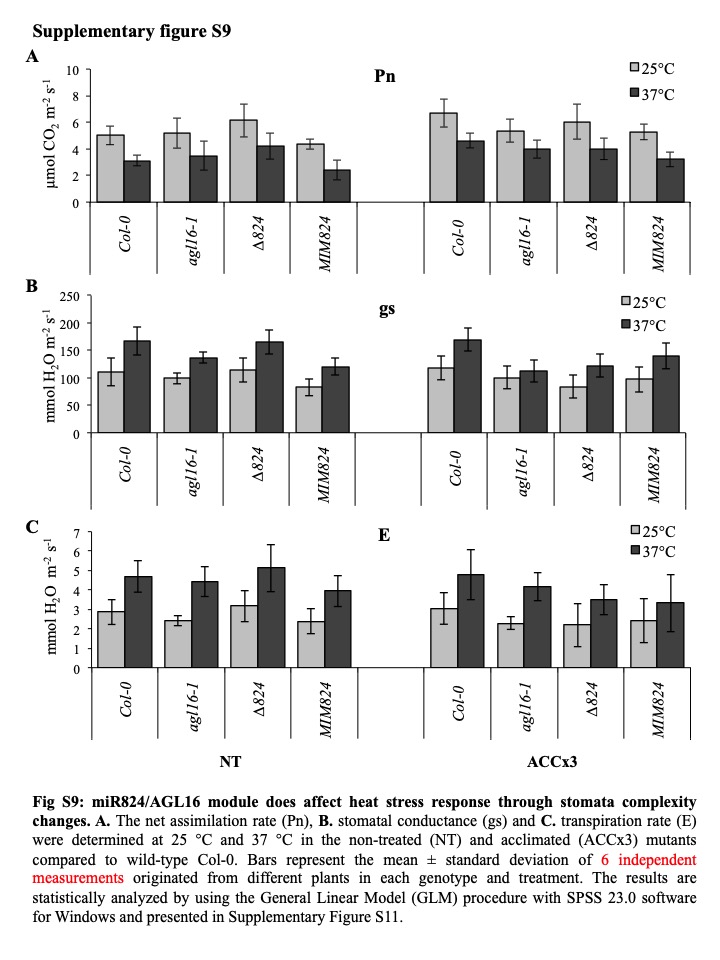

Supplement: Supplementary file 10 [file Image_9.jpeg]

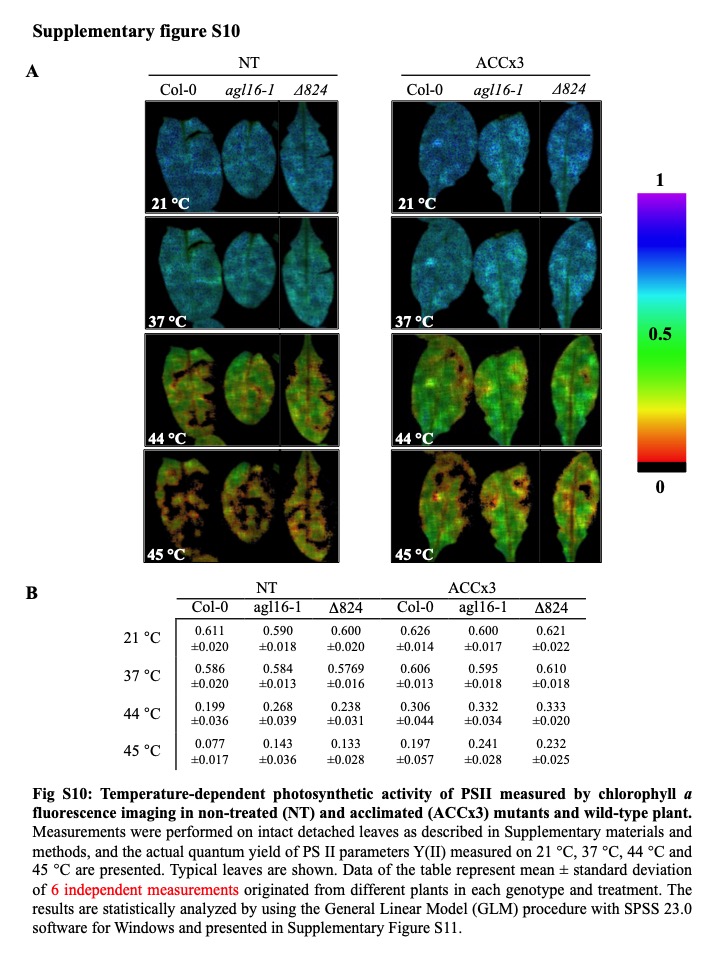

Supplement: Supplementary file 11 [file Image_10.jpeg]

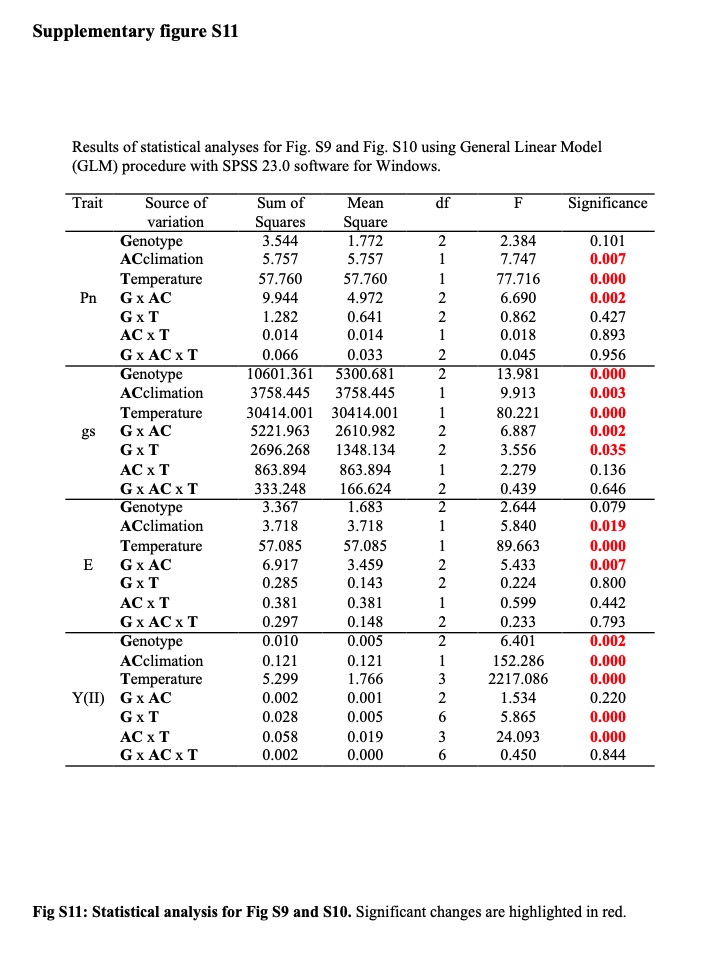

Supplement: Supplementary file 12 [file Image_11.jpeg]

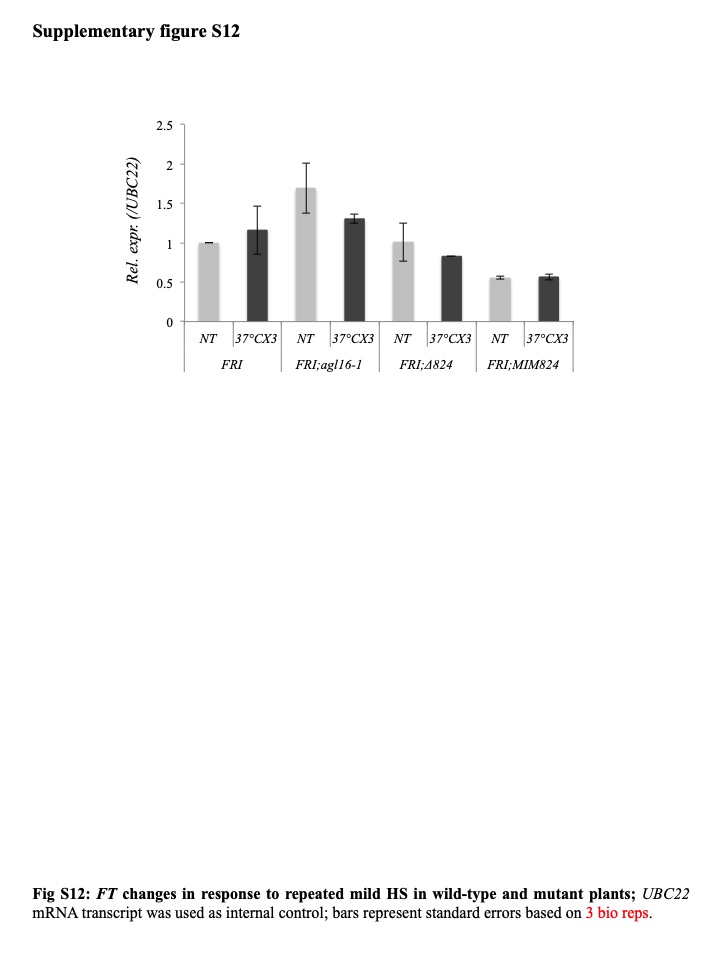

Supplement: Supplementary file 13 [file Image_12.jpeg]

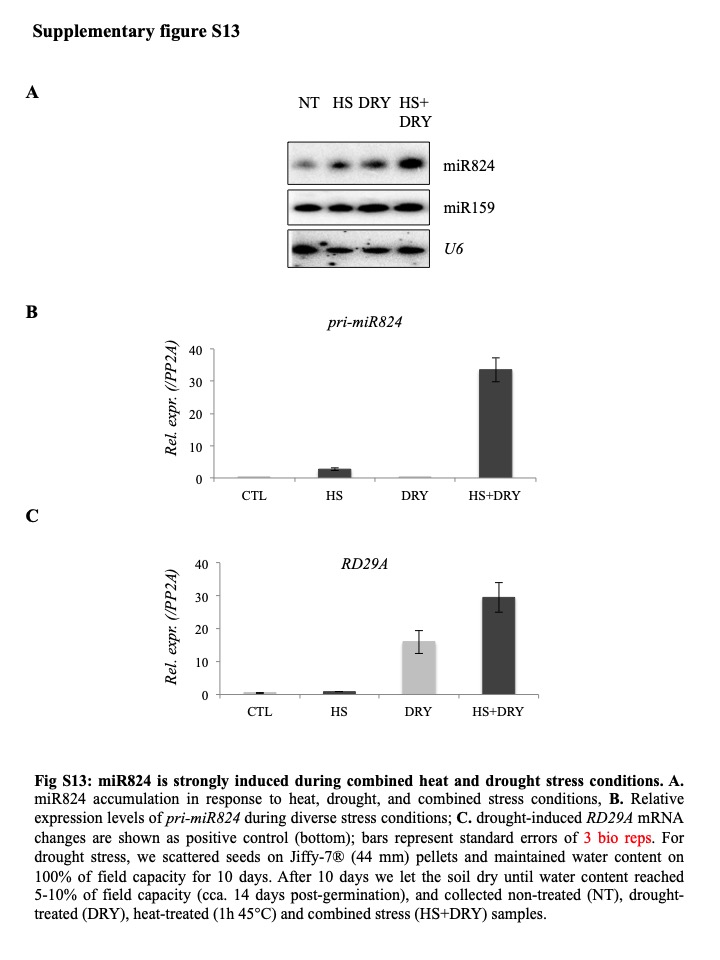

Supplement: Supplementary file 14 [file Image_13.jpg]

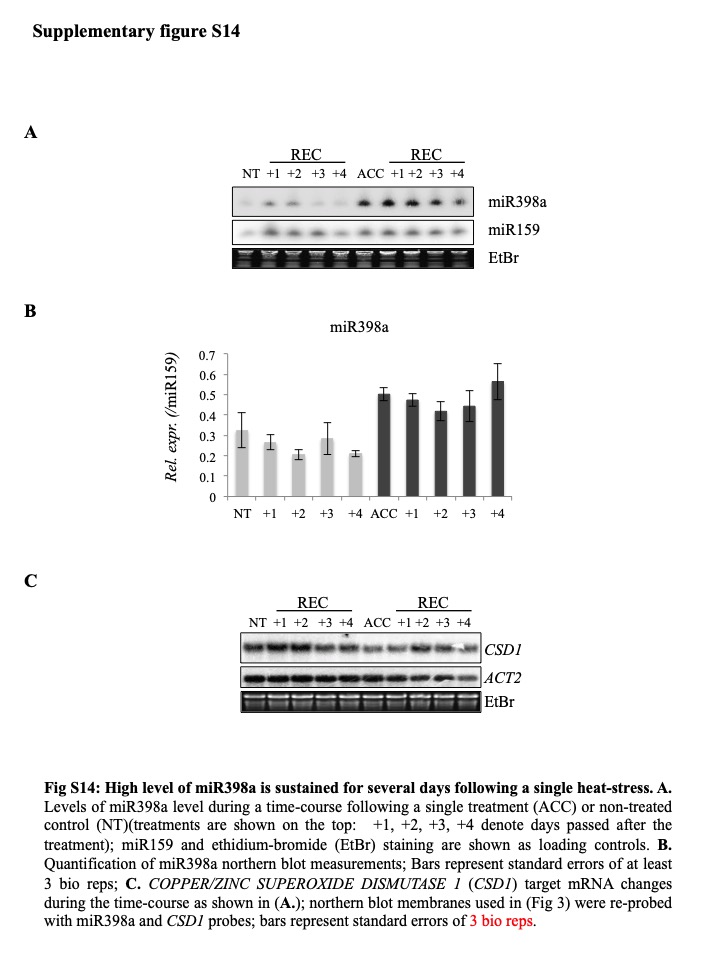

Supplement: Supplementary file 15 [file Image_14.jpeg]
